# Breeding progress of nitrogen use efficiency of cereal crops, winter oilseed rape and peas in long-term variety trials

**DOI:** 10.1007/s00122-023-04521-9

**Published:** 2024-02-08

**Authors:** F. Laidig, T. Feike, C. Lichthardt, A. Schierholt, H. P. Piepho

**Affiliations:** 1 Institute of Crop Science, Biostatistics Unit, University of Hohenheim, Fruwirthstrasse 23, 70599 Stuttgart, Germany; 2 Julius Kühn Institute – Federal Research Centre for Cultivated Plants, Institute for Strategies and Technology Assessment, Stahnsdorfer Damm 81, 14532 Kleinmachnow, Germany; 3 Bundessortenamt, Osterfelddamm 60, 30627 Hannover, Germany; 4 Plant Breeding Methodology, Georg-August-University Göttingen, Carl-Sprengel-Weg 1, 37075 Göttingen, Germany

## Abstract

**Key message:**

Grain yield and NUE increased over time while nitrogen yield did not drop significantly despite reduced nitrogen input. Selection for grain and nitrogen yield is equivalent to selection for NUE.

**Abstract:**

Breeding and registration of improved varieties with high yield, processing quality, disease resistance and nitrogen use efficiency (NUE) are of utmost importance for sustainable crop production to minimize adverse environmental impact and contribute to food security. Based on long-term variety trials of cereals, winter oilseed rape and grain peas tested across a wide range of environmental conditions in Germany, we quantified long-term breeding progress for NUE and related traits. We estimated the genotypic, environmental and genotype-by-environment interaction variation and correlation between traits and derived heritability coefficients. Nitrogen fertilizer application was considerably reduced between 1995 and 2021 in the range of 5.4% for winter wheat and 28.9% for spring wheat while for spring barley it was increased by 20.9%. Despite the apparent nitrogen reduction for most crops, grain yield (GYLD) and nitrogen accumulation in grain (NYLD) was increased or did not significantly decrease. NUE for GYLD increased significantly for all crops between 12.8% and 35.2% and for NYLD between 8% and 20.7%. We further showed that the genotypic rank of varieties for GYLD and NYLD was about equivalent to the genotypic rank of the corresponding traits of NUE, if all varieties in a trial were treated with the same nitrogen rate. Heritability of nitrogen yield was about the same as that of grain yield, suggesting that nitrogen yield should be considered as an additional criterion for variety testing to increase NUE and reduce negative environmental impact.

**Supplementary Information:**

The online version contains supplementary material available at 10.1007/s00122-023-04521-9.

## Introduction

Nitrogen is an essential nutrient in crop production and human nutrition. Prior to the industrial production of synthetic nitrogen through the Haber–Bosch process, nitrogen was the limiting factor in agro-ecosystems and for food production as a whole (Rütting et al. 2018). The great success of the Green Revolution was largely driven by the availability of mineral fertilizers, especially nitrogen (Erisman et al. 2008), and by the parallel development of high-yielding fertilizer-responsive varieties (Borlaug 1968). Those were able to use higher nitrogen fertilizer rates more efficiently for the production of higher yields. The tremendous increase of global food production in the past was possible mainly by increasing the input of nitrogen fertilizer (Stewart et al. 2005). In Western and Central Europe, synthetic fertilizer N application in kg ha^−1^ increased until 1990 and then steadily decreased while crop N removal in kg ha^−1^ increased (Cassman and Dobermann 2022; Einarsson et al. 2021). Today 50% of the nitrogen produced synthetically by the Haber–Bosch process is used for the three major cereals, i.e., maize (16%), rice (16%) and wheat (18%). Those grain crops cover the majority of human food calories and proteins consumed either directly as grain or indirectly through livestock (Cassman and Dobermann 2022).

**Fig. 1:**
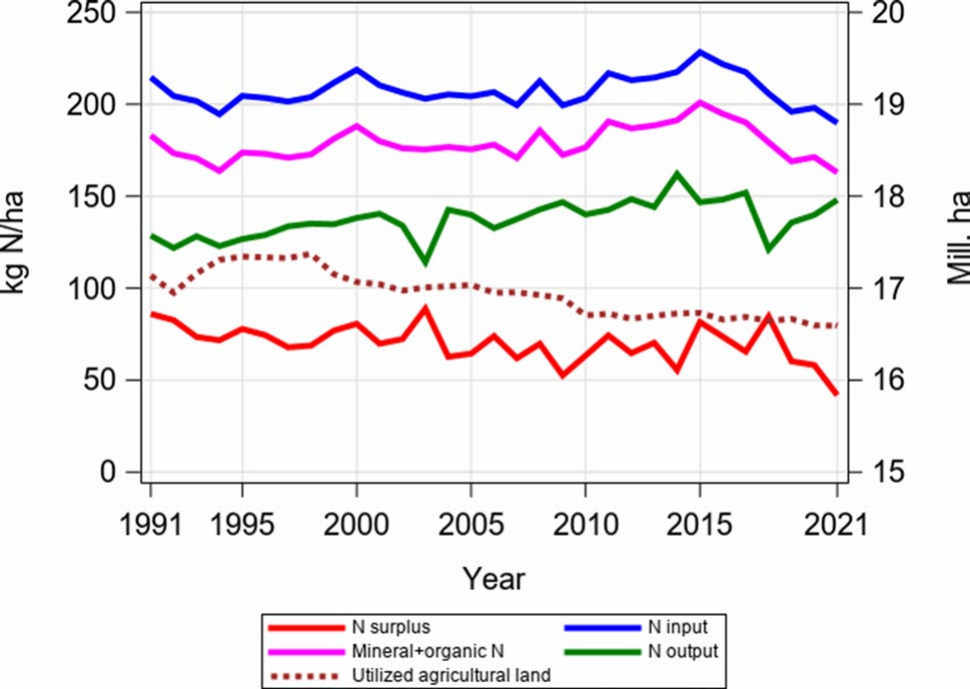
Development of total nitrogen input, N from mineral and organic fertilizer, N output and N surplus in kg ha^−1^ for Germany 1991–2021, and area of utilized agricultural land in Mill. ha. Source https://www.bmel-statistik.de/landwirtschaft/tabellen-zur-landwirtschaft#c8262, SJT-3070400-0000.xlsx Landwirtschaftlich genutzte Fläche nach Kulturarten. https://www.bmel-statistik.de/landwirtschaft/tabellen-zur-landwirtschaft#c8273, MBT-0111260-0000 Nährstoffbilanz insgesamt von 1990 bis 2021 in kg N/ha

In Germany, the reported total nitrogen input per hectare (ha) which consists of mineral and organic N fertilizer, N deposition and biological N fixation as well as N from seeds and planting material was quite stable since the 1990s, but recently shows a decreasing trend from 2015 onwards (Fig. 1). At the same time, the total output of N in the harvested plant material slightly increased between 1991 and 2021, while the N surplus budget and the area of utilized agricultural land decreased slightly. The average N input in 2017–2021 was 201 kg N ha^−1^, of which the N input from mineral and organic N was 174 kg N ha^−1^. For the same period, the average annual N output was 139  kg N ha^−1^ and the N surplus budget 62 kg N ha^−1^, which corresponds to an input–output efficiency of 69% and a surplus budget of 31% of total N input (BMEL 2022a, b), respectively. We should note that these figures are aggregated over all German regions. The highest surplus was reported in the northwestern and the southeastern German regions. Häußermann et al. (2020) reported slightly differing numbers for N budgets in Germany, i.e., that 46% of total N input stem from mineral fertilizer, 42% from organic fertilizer, 6% from biological N fixation and 6% from atmospheric deposition in 1995–2017. Unfortunately, reliable statistical data on mineral fertilizer sales are published only for Germany as a whole. From the fact that nitrogen statistics in Fig. 1 are the average over arable and grassland, it can be assumed that significantly more nitrogen was applied in arable land than reflected by the average. In addition, there are so far no publicly accessible statistics available regarding on-farm N fertilization rates for different crops.

The nitrogen surplus lost to the air, surface water and groundwater causes serious environmental damage. Lassaletta et al. (2014) reported that on global scale more than half of the N added to cropland is lost to the environment. While high-resolution data on the nitrogen use efficiency (NUE) of crop production, i.e., the ratio of grain produced per unit of available nitrogen in the soil (Moll et al. 1982), are lacking for most countries worldwide, few studies aimed to provide estimates of global NUE. As such, Raun and Johnson (1999) reported that NUE was approximately 33% in worldwide cereal production. In comparison, Cassman and Dobermann (2022) recently reported a global NUE of 40–50%.

Improving NUE and reducing N surplus is crucial for reducing the negative impacts on water quality as well as riparian and aquatic ecosystems in Germany and globally (Cassman and Dobermann 2022). With nitrogen being the major source of greenhouse gas emissions of crop production, an increased NUE would also help to reduce nitrogenous gases and their contribution to climate change (Riedesel et al. 2022). The reduction of N surplus needs to be realized without comprising global food security, where N fertilizer is an indispensable input. According to Connor (2008), only half of the current world population could be fed without synthetic mineral fertilizer.

Despite the above-mentioned rather low NUE in crop production, NUE increased in several countries across relevant crops (e.g., Ladha et al. 2016; Lassaletta et al. 2014). Furthermore, different studies report considerable breeding progress in NUE in winter wheat (Cormier et al. 2016; Sieling and Kage 2021; Ivic et al. 2021; Hawkesford and Riche 2020), triticale (Neuweiler et al. 2022), spring barley (Bingham et al. 2012) and oilseed rape (Kessel et al. 2012; Bouchet et al. 2016; Stahl et al. 2019, 2017).

NUE is a complex trait with many contributing processes. Apart from the type, amount and timing of fertilization, it is the result of the interaction between soil, weather, genotype and management measures. In this study, we refer to the NUE definition given by Hawkesford and Riche (2020): “NUE is the yield of grain produced per unit of N available to the crop. It is expressed as kg N in grain per kg N available.” For a more detailed description of NUE and its components, see, e.g., Moll et al. (1982), Good et al. (2004) and Hawkesford and Riche (2020).

Diverse factors and their interactions influence soil available nitrogen. Yan et al. (2019) reported based on published experiments with ^15^N-labeled fertilizer that most of the N in small grain crops (63%) came from sources other than the current year’s fertilizer. Part of the fertilizer N applied is assimilated into the soil. In cereals, Ladha et al. (2016) estimated that between 10% and 40% of applied N is fixed in the soil through microbial biomass and crop residues during the season of application. In a near steady-state situation, this quantity is approximately balanced by the N released from soil organic matter through mineralization. Accordingly, plant available N not only depends on the applied nitrogen fertilizer rate but also on the available soil mineral N, which is affected by the N in crop residues, atmospheric N deposition, soil quality, moisture and temperature, as well as microbial activity (e.g., Capriel 2014; Hawkesford 2014; Pituello et al. 2016; Cormier et al. 2016).

To reduce nitrogen surplus, a number of policies have been launched at the European Union and national level over the last decades to monitor and minimize N pollution. The EU Farm-to-Fork-Strategy and national agricultural policy have the goal to reduce nitrogen surplus by at least 50% and nitrogen fertilizer use by at least 20% by 2030 (BMEL 2019; EU 2020). At the same time, global demand for food and non-food agricultural products is increasing continuously. The current world population of 8 billion people is expected to grow to nearly 10 billion by 2050 resulting in an increased demand for food and respective carbohydrates and protein to be produced on limited global cropland resources.

Against the background of the required reduction of nitrogen surplus and respective nitrogen fertilizer rates, and the need to feed a growing world population, crop varieties with improved NUE are needed in combination with adapted crop management (e.g., Stahl et al. 2019; de Oliveira Silva et al. 2020). Several studies reported breeding progress in terms of yield (e.g., Laidig et al. 2014; Mackay et al. 2011; Voss-Fels et al. 2019) and disease resistance (e.g., Laidig et al. 2021, 2022; Zetzsche et al. 2020), while Cassman and Dobermann (2022) question whether new crop varieties with an apparent improvement in specific traits for NUE have actually emerged over the last decades. They attribute the improved NUE in high-fertilizer-use regions largely to a more judicious use of N fertilizers due to policies and regulations to reduce N use, rather than to the benefits of increasing crop yields.

In our study, we evaluate crops, whose protein concentrations have been routinely assessed in trials, i.e., for four cereal crops, winter oil seed rape and grain peas. Hence, the overall goals of this study are i) to quantify the breeding progress for NUE and related traits, ii) to investigate the question, whether high grain yield is closely linked to high grain nitrogen, (iii) to quantify the relationship between the varieties’ grain and nitrogen yield and the NUE of the grain and nitrogen yield, and (iv) to evaluate the potential for a direct selection for high grain nitrogen yield in trials. In particular, we first look at the relative frequency of pre-crops, the distribution of N rates and the soil-mineralized nitrogen at the beginning of the vegetation period and respective differences between crops. Secondly, we estimate the different crops’ long-term trends for breeding progress and determine changes in trends between 1995 and 2021. Thirdly, we compare the estimates of genotypic and environmental variation, correlation and heritability for crops and their traits.

## Materials and methods

### Variety trial and data

This study is based on data from official variety trials conducted by the Federal Plant Variety Office (Bundessortenamt, Hannover) for field crops at multiple locations during 1983 to 2021 to assess their value for cultivation and use. The investigated crops were winter wheat (WW), winter wheat under an organic testing regimen (WWORG), winter rye hybrid (WR Hyb) and population (WR Pop) varieties, spring wheat (SW), spring barley (SB), winter oil seed rape (WOSR) and grain peas (PEAS) (Table 1). On average, these crops accounted for 42.9% of the total arable land in Germany between 2019 and 2021. WW was the most important crop (24.7%), followed by WOSR (8.7%), WR (5.2%), SB (3.1%), PEAS (0.7%) and SW (0.5%) (BMEL 2022a). The trials were integrated in crop-specific continuous crop rotation regimen. The regular testing period for a newly applied variety was three years for WW, WWORG, WR, SW and SB, while for WOSR the regular testing period was two years between 1995 and 2010 and three years from 2011 onwards. Grain peas were tested for three years until 2006 and from then on for two years. Depending on the crop, time period and number of applied varieties, up to three parallel trial series (S1–S3) were run at each location in each year. In WW and SB, S1, S2 and S3 contain the varieties that were tested in their first, second and third year, respectively. In WR, varieties in their first and second testing year were tested together in one trial series and the varieties in their third year were tested separately. For PEAS, the varieties in the first and second and in SW and WWORG also those in the third test year were tested all together in one assortment.

**Table 1:** Overview on number of observations in varieties and trials

Crop	Code	First year	Observations	No. of trials	No. of varieties
Winter wheat	WW	1983	25,290	897	852
Winter wheat organic^a^	WWORG	2013	842	69	31
Winter rye hybrid varieties	WR Hyb	1989	7712	569	244
Winter rye population varieties	WR Pop	1989	2352	569	52
Spring wheat	SW	1983	9546	640	155
Spring barley	SB	1983	18,619	836	738
Winter oil seed rape	WOSR	1995	25,655	696	797
Grain peas	PEAS	1985	8769	533	277

^a^Winter wheat under organic testing regimen

For all crops, except WWORG and PEAS, up to three different treatment intensities with different N rates were applied until 1991, while from 1992 onwards, only two intensities with different N rates were applied. From 2005 on, both intensities received identical N rates. WWORG, WOSR and PEAS were tested under only one treatment. At least three reference varieties were included in each series. The references were included in each trial series and updated on a regular basis, ensuring at least partial overlap of sets of references used in successive years.

In WOSR and PEAS, grain protein concentration (GPC) and grain oil concentration (GOC) were assessed routinely at each location and each trial. For the cereals, GPC was assessed only at a subset of six to eight locations within a trial series for which grain quality samples were taken for testing baking and malting quality of varieties. GPC was derived from N assessed by near-infrared spectroscopy using the protein equivalent factor c = 5.7 for WW, WWORG and SW, and c = 6.25 for the other crops (DIN EN 15948, 2012). As only GPC was available in the dataset, nitrogen yield (NYLD) was calculated as N in kg ha^−1^ accumulated in grain by NYLD = 100 × GYLD × (DM/100)  × (GPC/100)/c), where DM is the percent dry matter content in grain and c is the protein equivalent factor.

**Fig. 2:**
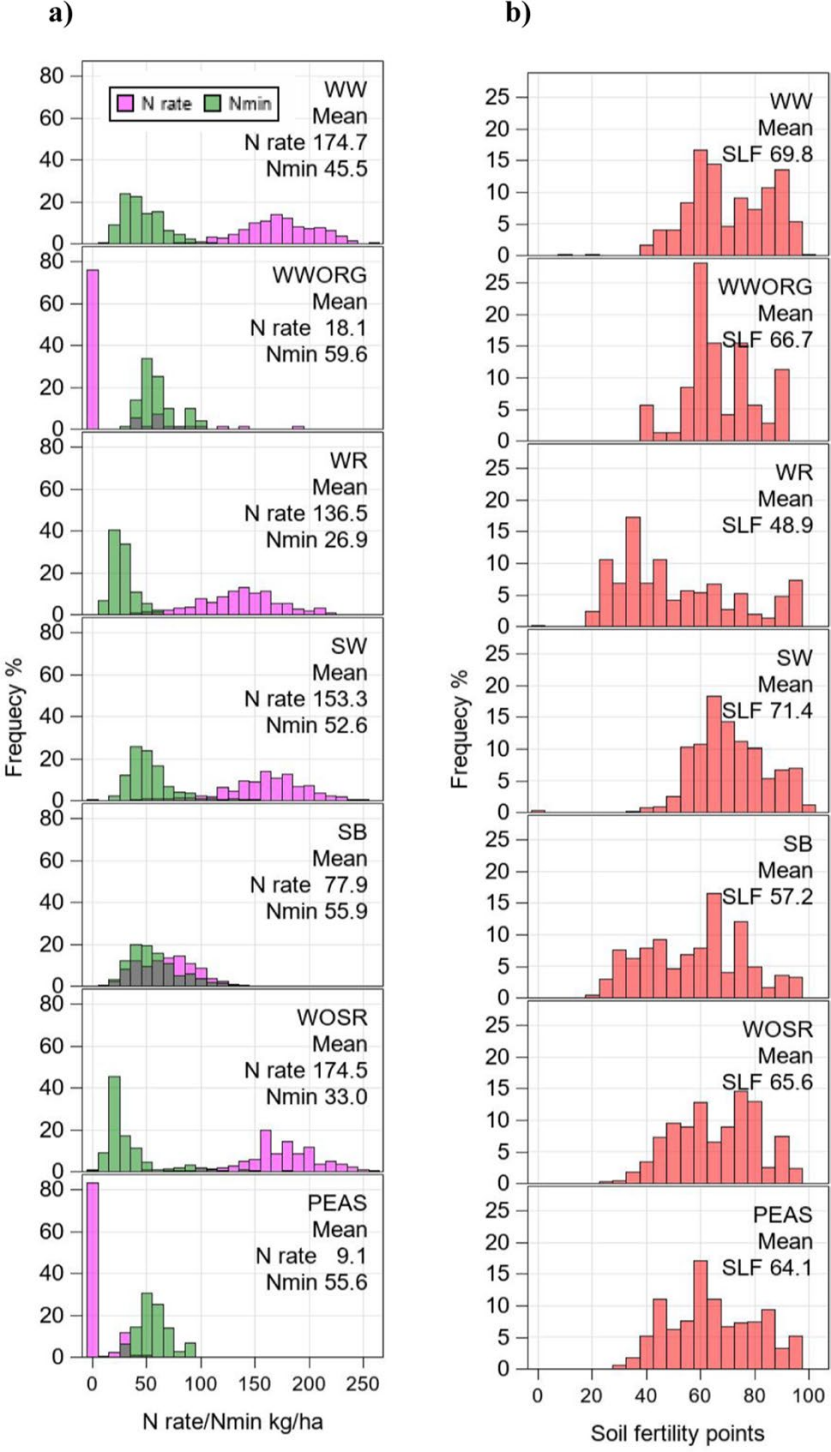
Frequency distribution based on years 1995–2021 of (**a**) nitrogen fertilization rates (N rate) for trials and predicted soil-mineralized nitrogen (Nmin) by Eq. (9) before beginning of vegetation period in harvest year and of (**b**) soil fertility points (SLF). *WW* winter wheat, *WWORG* winter wheat under organic testing regimen, *WR* winter rye, *SW* spring wheat, *SB* spring barley, WSOR winter oil seed rape, *PEAS* grain peas

Trials for all crops were managed by the regimen of good local agronomic practice, including the application of fertilizer and growth regulators as well as the control of pests and diseases. The recorded N fertilization rates (henceforth referred to as “N rate”) for each individual trial were accumulated as total nitrogen applied in kg ha^−1^. If organic fertilizer was applied, the N equivalent was taken into account and added to the applied mineral N quantity accordingly. In Fig. 2a, the distribution and the average N rates (magenta color) and predicted Nmin values (green color) 1995–2021 are shown, indicating considerable differences between crops and a large variability between trials within crops. Unfortunately, no data on plant available mineralized nitrogen in the soil (Nmin) was available until 2018. Hence, we predicted these missing data by using the available Nmin data in 2019–2021 (see section “Prediction of soil-mineralized nitrogen (Nmin)”). The total available nitrogen per trial is then the sum of N rate and Nmin. We evaluated three measures of NUE: NUE for grain yield (GYLD_NUE_), NUE for oil yield (OYLD_NUE_) and NUE for nitrogen yield (NYLD_NUE_) in grain, expressed as kg grain, kg oil and kg N per kg available N, respectively. NUE for PEAS was not considered in this study, because this crop usually received no N fertilizer, except at a very low rate as starter in spring; therefore, we did not evaluate NUE for GYLD and NYLD.

Trials were laid out as split-plot designs with main plots arranged in complete blocks. Trials with only one intensity were designed as complete blocks. Subplots within main plots were either laid out as randomized complete blocks or according to an alpha-lattice design. The harvested plot size was about 10 m^2^ on average. WR hybrid and WR population varieties were grown in the same trial and treated identically. Nevertheless, we analyzed both variety types separately. In WOSR, line and hybrid varieties were analyzed together, as only 30% of all tested varieties were line varieties and no single line variety has been registered since 2014. Compared to the large yield difference in winter rye between hybrids and population varieties, where heterosis can be utilized very effectively due to two divergent gene pools, the differences between lines and hybrids are relatively small in WOSR as shown, e.g., in Stahl et al. (2017). Accordingly, we decided that a separation is not meaningful in the context of this study.

We used only data from varieties tested for at least two years to achieve a good representation of the trial conditions. Data included in this study are shown in Table 1. The dataset was highly non-orthogonal with respect to variety × year combinations, whereas the variety × location combinations were orthogonal within year and trial series, i.e., all varieties were grown together at all locations within the same year and trial series. The data were checked for recording errors and outliers by calculating standardized residuals based on Eq. (1). We excluded observations with standardized residuals greater than ± 5.0 from further analysis.

### Soil fertility and pre-crops

Variety trials were conducted in the crops’ typical growing regions across Germany. For each trial, soil fertility points (In German: Ackerzahl) were recorded. Soil fertility describes the site-specific productiveness of arable land in Germany (Weiser et al. 2018) and it is the most important factor describing the natural yield potential of a trial site (Laidig et al. 2022). Soil fertility and respective yield potential, however, is not only dependent on soil type, geological age of the parent rock and soil development stage, but also influenced by factors like climate, temperature, precipitation and topography. In a field rating, soil fertility is assessed by taking into account natural environmental conditions of a specific area of arable land (BodSchätzG 2007; Blume et al. 2015, Chap. 11.2, p. 564 ff). In Germany, soil fertility is graded on a scale from 1 to 120 points, where 1 means very poor and 120 very good soil fertility. In Fig. 2b, we show the distribution of soil fertility points for the different crops assessed in our study.

**Fig. 3:**
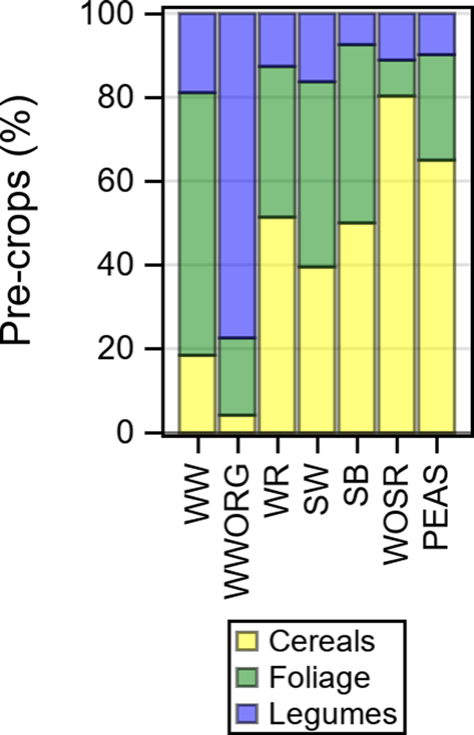
Pre-crops as percent of total number of trials. Observations are based on individual trials (year × location × trial series combinations) from 1995 to 2021. Pre-crops were categorized as cereals, foliage (e.g., sugar beet, oil seed rape and maize) and legumes (e.g., beans, peas and clover). *WW* winter wheat, *WWORG* winter wheat under organic testing regimen, *WR* winter rye, *SW* spring wheat, SB spring barley, *WOSR* winter oil seed rape, *PEAS* grain peas

The specific pre-crops affect growth and yields of the succeeding crop. Legumes are generally more beneficial than, e.g., cereals or foliage crops. We categorized pre-crops into three groups: cereals, foliage crops (e.g., sugar beet, oil seed rape and maize) and legumes (e.g., beans, peas and clover). The percentage share of groups is shown in Fig. 3. In WW, foliage crops were the most frequent pre-crop with more than 60%, while in WOSR cereals predominated with 80%. In WWORG, legumes were grown as pre-crops in more than 80% and foliage crops in about 15% of trials.

### Statistical analysis

#### Basic Model

For a given observation (average over replications), we used a model with factors genotype, location, trial series and year and considering linear genetic and quadratic non-genetic long-term trends given by

1 $${y}_{ijkl}=\mu +{\beta {r}_{i}+{\gamma }_{1}{t}_{k }+{\gamma }_{2}{t}_{k}^{2}+ G}_{i}+{L}_{j}+{Y}_{k}+{\left(LYT\right)}_{jkl}+{\left(GL\right)}_{ij}+{\left(GY\right)}_{ik}+{\epsilon }_{ijkl},$$

where *y*_*ijkl*_ is the mean yield of the *i*th genotype in the *j*th location and *k*th year within the *l*th trial series, *μ* is the overall mean, *β* is a fixed regression coefficient for the genetic trend, *r*_*i*_ is the first year in trial of the *i*th genotype, ${\gamma }_{1}$ and ${\gamma }_{2}$ are fixed linear and quadratic regression coefficients for the non-genetic trend, *t*_*k*_ is the covariate for the *k*th calendar year, *G*_*i*_ is the main effect of the *i*th genotype, *L*_*j*_ is the main effect of the *j*th location, *Y*_*k*_ is the main effect of the *k*th year, *T* indicates the trial series (S1, S2, S3) and (*LYT*)_*jkl*_ is the effect of the *l*th trial series within the *jk*th location × year combination, (*GL*)_*ij*_ is the *ij*th genotype × location interaction effect, (*GY*)_*ik*_ is the *ik*th genotype × year interaction effect and ${\epsilon }_{ijkl}$ is a residual comprising the genotype × location × year interaction ${\left(GLY\right)}_{ijk}$, the genotype × location × year × trial series interaction ${\left(GLYT\right)}_{ijkl}$ and the error of a mean arising from sampling the replications. We confounded ${\left(GLY\right)}_{ijk}$ and ${\left(GLYT\right)}_{ijkl}$ with the residual error, because they were only based on the few reference varieties and were of about the same magnitude as the residual without these interactions (Hartung et al. 2023). All effects, except *μ*, *β* and *γ*, are assumed to be random and independent with constant variance for each effect. We modeled the genetic trend by linear and the non-genetic trend by quadratic regression terms, because inspection of graphical representation of trends indicated that genetic trends were approximately linear and non-genetic trends of quadratic shape. We estimated variance components for the random effects in Eq. (1) to get insight on the relative impact of genotypes and environmental factors on total variation by restricted maximum likelihood method (REML). We included a genetic and a non-genetic trend in Eq. (1) to avoid inflated variance components for the genotypic and year effects in case that these effects were subject to time trends. Hence, both effects can be interpreted as deviations from their respective trend functions.

#### Estimation of heritability

Traits assessed in registration trials should be useful for evaluating the value for the cultivation and use of a variety. Further, they should also have a reasonable predictive power to proof their performance also on farmers’ fields. Therefore, we estimated broad sense heritability for cycle means of varieties. A testing cycle includes all trials of a variety's two- or three-year testing cycle. We used equation

2a $${H}^{2}={\sigma }_{G}^{2}/\left({\sigma }_{G}^{2}+\frac{{v}_{d}}{2}\right),$$

where ${\sigma }_{G}^{2}$ is the genotypic variance component and *v*_*d*_ is the average variance of a difference between the means of two varieties tested in the same cycle (Piepho and Möhring 2007). To obtain ${v}_{d}$, a dummy dataset was generated having the exact same structure in terms of the number of trials in each of the three or two years of a cycle, including the overlap of locations used in more than one year (Table 2). Dummy observations were generated for the responses of two varieties tested for three years. The dataset was analyzed using the GLIMMIX procedure of SAS, plugging on the variance component estimates of all random effects in Eq. (1) and fixing these during analysis. As the variances were fixed, the values of the dummy response were immaterial (Piepho et al. 2022). The variety main effect was taken as fixed, so adjusted means could be computed, as well as the variance of the difference between the two means (*v*_*d*_).

**Table 2:** Heritability *H*^2^ of traits based a crop’s testing cycle (Eq. 2a) and heritability based on an individual trials *q*^2^ (Eq. 2b), where *n* corresponds to total number of trials per testing cycle, *n*_1_, *n*_2_, *n*_3_ to trials in the first second and third testing year, *n*_12_, *n*_13_, *n*_23_ to number of overlapping locations

	Number of trails per cycle							NYLD		GPC/GOC		GYLD/OYLD		NYLD_NUE_		GYLD/OYLD_NUE_	
Crop	*n*	*n*_1_	*n*_2_	*n*_3_	*n*_12_	*n*_13_	*n*_23_	*H*^2^	*q*^2^	*H*^2^	*q*^2^	*H*^2^	*q*^2^	*H*^2^	*q*^2^	*H*^2^	*q*^2^
Winter wheat	24	8	8	8	0	0	4	0.83	0.22	0.96	0.65	0.90	0.41	0.82	0.21	0.90	0.39
Winter wheat organic	22	7	7	8	5	6	6	0.61	0.14	0.95	0.68	0.83	0.37	0.56	0.12	0.77	0.29
Winter rye Hyb	24	8	8	8	0	0	6	0.61	0.15	0.89	0.48	0.65	0.19	0.54	0.11	0.63	0.17
Winter rye Pop	24	8	8	8	0	0	6	0.72	0.18	0.85	0.35	0.82	0.28	0.67	0.16	0.82	0.30
Spring wheat	21	7	7	7	5	6	5	0.74	0.30	0.90	0.58	0.62	0.21	0.69	0.26	0.59	0.19
Spring barley	23	8	8	7	0	0	3	0.57	0.08	0.78	0.20	0.72	0.21	0.56	0.07	0.72	0.16
Winter oil seed rape	36	9	13	14	0	1	7	0.85	0.25	0.94	0.49	0.80	0.15	0.84	0.23	0.79	0.20
Winter oil seed rape^a^										0.95	0.58	0.77	0.20			0.79	0.20
Grain peas	20	10	10		7			0.88	0.27	0.96	0.57	0.88	0.28				
Mean	24.3	8.1	8.6	8.6	2.1	1.9	5.3	0.73	0.20	0.91	0.51	0.78	0.26	0.67	0.17	0.75	0.24

All results are based on years 1995–2021. Grain peas were tested for only two years. NUE for grain peas were not listed as they received no regular nitrogen fertilizer

*Organic* Winter wheat under organic testing regimen with no mineral N and no pesticides, *Hyb* hybrid varieties, *Pop* population varieties, *NYLD* nitrogen yield, *GPC/GOC* grain/oil protein concentration, *GYLD/OYLD* grain/oil yield, *NYLD*_NUE_, *GYLD/OYLD*_NUE_ nitrogen use efficiency for NYLD and GYLD/OYLD, respectively

^a^Grain oil concentration (GOC), Oil yield (OYLD) and NUE for oil yield (OYLD_NUE_) in winter oilseed rape

Heritability *H*^2^ estimated by Eq. (2a) depends on the relative magnitude of variance components but also on the number of years and locations typical for a specific crop’s testing cycle. To get a measure of heritability independent of the number of locations and years and hence comparable between crops, we estimated a trial-specific measure, given by

2b $${q}^{2}={\sigma }_{G}^{2}/\left({\sigma }_{G}^{2}+ {\sigma }_{GY }^{2}+{\sigma }_{GL }^{2}+ {\sigma }_{Res }^{2}\right),$$

where ${\sigma }_{G}^{2}$, ${\sigma }_{GY}^{2}{, \sigma }_{GL }^{2}$ and ${\sigma }_{Res }^{2}$ are variance components for genotypes, the interaction of genotypes × year, genotypes × location and the residual error.

#### Model for overall trend

The overall trend was modeled by confounding year and genotypes within years, i.e., genotypes are nested within years (Laidig et al. 2014). Thus, compared with the basic model (Eq. 1), for this analysis we dropped effects involving genotypes that are not nested within years, i.e., the effects ${G}_{i}$ and (*GL*)_*ij*_. Then the reduced model is given by

3 $${y}_{ijkl}=\mu + {L}_{j}+{Y}_{k}+{\left(LYT\right)}_{jkl}+{\left(GY\right)}_{ik}+{\left(GLYT\right)}_{ijkl}.$$

Further, we assumed that *Y*_*k*_ is subject to an overall long-term time trend, confounding genetic and non-genetic trends. We extended Eq. (3) by fixed linear and quadratic regression coefficients. Then, the model is given by

4 $${y}_{ijkl}=\mu +{\alpha }_{1}{t}_{k }+ {\alpha }_{2}{t}_{k}^{2}+ {L}_{j}+{Y}_{k}+{\left(LYT\right)}_{jkl}+{\left(GY\right)}_{ik}+{\left(GLYT\right)}_{ijkl}.$$

where ${\alpha }_{1}$ and ${\alpha }_{2}$ are fixed linear and quadratic regression coefficients for the overall trend, ${t}_{k}$ is the continuous covariate for the calendar year. The expected value under this model is given by

5 $$E\left({y}_{ijkl}\right)=\mu +{\alpha }_{1}{t}_{k }+ {\alpha }_{2}{t}_{k}^{2}.$$

#### Estimation of breeding progress

As WOSR data were only available from 1995 onwards, we estimated the change achieved between ${t}_{k}=$ 1995 and 2021 based on the overall trend given by Eq. (4) to ensure that estimation of breeding progress between crops and traits was based on the same time period. Hence, for all crops we calculated the change as the difference of the predicted values for year 2021 and 1995 by

$${\text{Diff}}=E\left({y}_{ijkl}\left|{t}_{k}=2021\right.\right)-E\left({y}_{ijkl}\left|{t}_{k}=1995\right.\right)=$$

6 $${\alpha }_{1} \left(2021-1995\right)+{\alpha }_{2}\left({2021}^{2}-{1995}^{2}\right)\,\mathrm{ using\ Eq}. (4).$$

#### Genotypic, environmental and G × E correlation

As in long-term trials, the strength of the association between pairs of traits can be influenced by several effects. Simple correlation coefficients over all observations, for example the Pearson sample correlation coefficient, are not always appropriate to allow valid inferences as the structure of the trial series is not considered. The simple sample correlation coefficient does not indicate which effect, genotype or environment, was dominating. To get insight, we therefore decomposed the correlation between traits by their individual random effects as given in Eq. (1). We estimated correlation coefficients (Piepho 2018) between traits based on variety × year × location × trial series observations (Eq. 1).

The correlations between random effects of Eq. (1) were calculated assuming a multivariate model with traits as independent variables. We choose a univariate approach from which correlations for pairs of traits can be inferred (Piepho et al. 2014):

1. We calculated variance components of random effects according to the model of Eq. (1) for trait *U* and *V* and for the difference $U-V$ between both traits.
2. We computed covariances between the random effects of trait *U* and *V* from variance components obtained from univariate models by using the equation
  7 $${\text{var}}(U-V)=\mathrm{ var} (U) +\mathrm{ var }(V) -2\mathrm{cov }(U,V) \iff$$
  8 $$\mathrm{Cov }(U,V)=\frac{1}{2}({\text{var}}\left(U\right)+{\text{var}}\left(V\right)-{\text{var}}\left(U-V\right))$$
3. We used variances of random effects from Eq. (1) and their covariance from Eq. (7) to calculate the correlation coefficients.

The marginal correlation coefficient was derived by the marginal variances and covariances which are the sum over individual random effects of var ($$U$$), var (V) and cov ($$U,V)$$. Compared to the simple Pearson correlation coefficient (*r*_*P*_), the marginal correlation (*r*_*M*_) is the correlation on the level of observations (variety × year × location × trial series combinations), which takes into account the model structure of the trial series (Piepho 2018). We aggregated the variances and covariances of the random effects $${L}_{j},{Y}_{k}, {\left(LYT\right)}_{jkl}$$ to the environmental (E) effect and $${\left(GL\right)}_{ij},{\left(GY\right)}_{ik}$$ to the genotype × environment interaction (G × E) effect. Finally, we obtained the correlation coefficients for the marginal (*r*_*M*_), genotypic (*r*_*G*_), the G × E (*r*_*G×E*_), the environmental (*r*_*E*_) and the residual effects (*r*_*Res*_). The magnitude of the marginal correlation depends mostly on the magnitude of the random effect with the largest variance and covariance components.

#### Prediction of soil-mineralized nitrogen (Nmin)

Soil-mineralized nitrogen was assessed up to 60 cm soil depth for each trial in spring before the start of vegetation. Nmin was considered by fixing the target N rate for a given trial such that available N is the sum of applied N rate and the assessed Nmin (DUEV 2017). However, Nmin data were only available for 2019–2021. We utilized those available data (across crops, *n* = 259) to predict Nmin data for trials where no data were available. As the distribution of Nmin values showed a right-skewed shape, we transformed the data by a logarithmic function to achieve a more symmetric distribution. The model is given by

9 $${y}_{ijkl}=\mu + {(CP)}_{im}+{\delta }_{m}{a}_{jkl}+{\eta }_{i}{c}_{jkl}+{L}_{j}+{Y}_{k}+{\left(LYT\right)}_{jkl},$$

where *y*_*ijkl*_ is the log-transformed Nmin assessed for the *i*th crop at the *l*th trial series within the *j*th location and *k*th year, *μ* is the overall mean, (*CP)*_*im*_ is a categorial effect of the *i*th crop and the *m*th pre-crop, ${\delta }_{m}$ the fixed pre-crop-specific regression coefficient of the linear trend for SLF, *a*_*jkl*_ is the covariate represented by the SLF point of the *jkl*th trial and ${\eta }_{i}$ the fixed crop-specific regression coefficient of the linear trend for the N rate and *c*_*jkl*_ is the covariate represented by the N rate in kg ha^−1^ of the *jkl*th trial, *L*_*j*_ is the main effect of the *j*th location, *Y*_*k*_ is the main effect of the *k*th year and ${\left(LYT\right)}_{ijkl}$ is the residual error. We assumed that the effects *L*_*j*_, *Y*_*k*_ and (*LYT*)_*jkl*_ are random and independent with constant variance, while all other effects are considered as fixed. In the model selection procedure we started with a basic model which was given by the random effects ${L}_{j}$, ${Y}_{k}$ and ${\left(LYT\right)}_{jkl}$, only, and added stepwise the fixed effects as given in Eq. (9). As selection criterion we used the coefficient of determination *R*^2^ (Piepho 2019). We stopped the selection of model terms until it reached *R*^2^ = 41.5% and could not be improved further. The resulting model is given by Eq. (9). The back-transformed best linear unbiased predictors (BLUP) for Nmin of Eq. (9) were used for all trials between 1983 and 2021, assuming that the Nmin was not subject to a time trend in 1983–2021.

## Results

### Overall trends for breeding progress of NUE and related traits

**Fig. 4:**
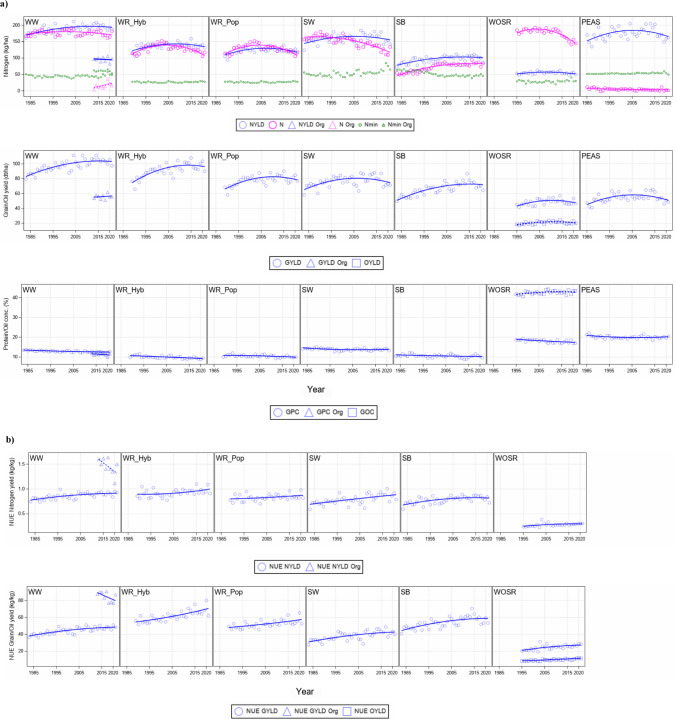
Adjusted overall year means (Eq. 3) (blue circles), quadratic regression lines (Eq. 5) (blue lines) based on years 1983–2021 (**a**) for nitrogen yield in grain (kg N ha^−1^), nitrogen fertilizer application rate (kg ha^−1^) (magenta circles and lines) and average predicted soil-mineralized nitrogen (green horizontal lines), grain/oil yield (dt ha^−1^), grain/oil protein concentration (%), (**b**) NUE for grain yield and nitrogen yield. NUE for grain peas were not displayed as they received no regular nitrogen fertilizer. *WW* winter wheat; *WWORG* winter wheat under organic testing regimen; *WR* winter rye, *Hyb* hybrid and *Pop* population varieties, *SW* spring wheat, *SB* spring barley, *WSOR* winter oil seed rape, *PEAS* grain peas, *NYLD* nitrogen yield in grain, *GPC* grain protein concentration, *GOC* grain oil concentration, GYLD grain yield, *OYLD* oil yield, *NUE GYLD*, *NUE NYLD*, *NUE OYLD* NUE for grain yield, nitrogen yield in grain, oil yield in grain

Generally, we should note that breeding progress estimated for all traits was subject to two confounded processes, the introduction of continously improved new varieties and decreasing N rates. Overall breeding progress was estimated by a mixed linear model where year effects were assumed to follow a quadratic time trend (Eqs. 4 and 5). The difference of the estimated trends in 2021 and 1995 was considered as the breeding progress (Eq. 6). In Table 3 the estimated levels in 1995 and 2021, plus the absolute and relative differences are shown for NYLD, N rate, GYLD/OYLD, GPC/GOC, and for NYLD_NUE_ and GYLD/OYLD_NUE_. In Fig. 4, trends and Nmin levels are displayed.

**Table 3:** Performance levels of overall trends at years 1995 and 2021 and difference between levels 2021 and 1995 expressed in absolute (Diff) and relative (%) values based on level 1995 (Eq. 6). Table for (**a**) nitrogen yield in grain kg ha^−1^, N fertilizer kg ha^−1^, grain protein concentration % and grain/oil yield dt ha^−1^ and (**b**) for nitrogen use efficiency for nitrogen yield kg kg^−1^ and grain yield/oil kg kg^−1^. NUE for grain peas were not listed as they received no regular nitrogen fertilizer

**(a)**																				
	Nitrogen yield kg ha^−1^					Nitrogen fertilizer kg ha^−1^					Grain/Oil protein concentration %					Grain/Oil yield dt ha^−1^				
Crop	1995	2021	Diff	%	Sign	1995	2021	Diff	%	Sign	1995	2021	Diff	%	Sign	1995	2021	Diff	%	Sign
Winter wheat	187.4	194.0	6.6	3.5	^ns^	180.1	170.3	−9.8	−5.4	^ns^	13.1	12.5	−0.6	−4.4	^**^	95.4	103.3	7.9	8.3	^**^
Winter wheat organic^a^	100.6	94.9	−5.7	−5.7	^ns^	8.9	23.1	14.2	158.8	^ns^	11.7	11.1	−0.6	−5.3	^ns^	55.9	57.2	1.3	2.3	^ns^
Winter rye Hyb	134.1	135.1	1.1	0.8	^ns^	130.4	107.8	−22.6	−17.3	^***^	10.5	9.3	−1.2	−11.2	^***^	84.9	96.6	11.6	13.7	^**^
Winter rye Pop	122.0	117.9	−4.1	−3.3	^ns^	130.4	107.8	−22.6	−17.3	^***^	10.8	10.0	−0.9	−8.1	^*^	74.4	78.4	3.9	5.3	^ns^
Spring wheat	160.9	155.0	−5.9	−3.6	^ns^	163.1	115.9	−47.2	−28.9	^***^	14.0	13.9	0.0	−0.3	^ns^	76.8	74.4	−2.3	−3.0	^ns^
Spring barley	94.3	102.3	8.0	8.5	^*^	68.6	83.0	14.3	20.9	^***^	10.8	10.3	−0.5	−4.5	^ns^	64.0	72.4	8.4	13.2	^**^
Winter oil seed rape	51.3	51.4	0.0	0.1	^ns^	176.9	143.5	−33.4	−18.9	^***^	18.8	17.4	−1.4	−7.6	^**^	44.8	48.6	3.8	8.5	^*^
Winter oil seed rape^b^											41.6	42.7	1.2	2.8	^*^	17.9	20.3	2.4	13.5	^*^
Grain peas	176.0	164.6	−11.3	−6.4	^ns^	5.5	3.0	−2.5	−45.1	^ns^	20.1	20.3	0.1	0.7	^ns^	54.7	50.1	−4.5	−8.3	^ns^

**(b)**										
	NUE Nitrogen yield kg kg^−1^					NUE Grain/Oil yield kg kg^−1^				
Crop	1995	2021	Diff	%	Sign	1995	2021	Diff	%	Sign
Winter wheat	0.84	0.91	0.07	8.0	^*^	43.0	48.5	5.5	12.8	^***^
Winter wheat organic^a^	1.76	1.31	−0.45	−25.5	^ns^	96.8	79.6	−17.2	−17.8	^ns^
Winter rye Hyb	0.89	1.01	0.12	13.0	^*^	56.7	71.9	15.2	26.8	^***^
Winter rye Pop	0.81	0.88	0.07	9.1	^ns^	49.4	58.4	9.0	18.2	^***^
Spring wheat	0.76	0.88	0.12	15.9	^*^	36.2	42.4	6.2	17.2	^*^
Spring barley	0.76	0.83	0.06	8.0	^ns^	51.9	58.9	7.0	13.5	^*^
Winter oil seed rape	0.25	0.30	0.05	20.7	^**^	20.9	27.3	6.4	30.8	^***^
Winter oil seed rape^b^						8.7	11.8	3.1	35.2	^***^

^a^Years 2013–2021; ^b^Oil

*Hyb* hybrid varieties, *Pop* population varieties, *Diff* absolute difference between levels at years 2021 and 1995 based on overall quadratic regression estimates (Eq. 6), *%* relative difference as percent of yield level 1995, *NUE* nitrogen use efficiency expressed as nitrogen yield or grain/oil yield per kg applied N fertilizer and soil-mineralized nitrogen, *Sign* significance level; ^*^Significant at 5% level; ^**^Significant at 1% level; ^***^Significant at 0.1% level

The N rate decreased in most crops, especially in WR, SW and WOSR. SB was the only crop where the N rate increased by 14.3 kg ha^−1^, corresponding to 20.9%. Highest N rates were applied in WW with 180.1 kg ha^−1^ in 1995 and 170.3 kg ha^−1^ in 2021 followed by WOSR with 176.9 kg ha^−1^ in 1995 and 143.5 kg ha^−1^ in 2021. WWORG and PEAS received only negligible mineral fertilizer so that their results are not directly comparable with the other crops.

In WWORG, no significant trends were estimated (Table 3) due to the large year-to-year variation relative to the short 9-year period. This is another reason for the limited comparability with the other crops which build on data from 27 years. Despite the low number of years, a rough comparison with WW indicated a slight nonsignificant increase in GYLD (2.3%) but on a GYLD and NYLD level of more than 50% below the corresponding levels in WW. This may be partially attributed to the lower recovery rate of organic N fertilizer compared to the mineral N fertilizer which was supported by Yan et al. (2019) reporting a higher recovery from mineral N (37%) than from organic N (27%). The lower recovery of organic N fertilizer may be partially compensated by the observed higher Nmin in WWORG (59.6 kg ha^−1^) compared to WW (45.5 kg ha^−1^) as about 80% of WWORG trials had legumes as pre-crops (Fig. 2).

The change for NYLD between 1995 and 2021 was in the range of −5.7% (WWORG) to 8.5% (SB), but only the increase in SB was significant, yet at a low NYLD level in 1995 (94.3 kg ha^−1^) and in 2021 (102.3 kg ha^−1^) We found the highest NYLD in WW and PEAS whereas WOSR had a rather low NYLD (about 51 kg ha^−1^).

In most crops, GPC decreased, especially in WR hybrid varieties (−11.2%). Noticeably, GPC in WOSR decreased (−7.6%) while GOC in WOSR increased slightly (2.8%). GYLD increased in the range of 2.3% (WWORG) and 13.7% (WR hybrid varieties). A nonsignificant decrease in GYLD was found in SW (−3.0%) and PEAS (−8.3%).

In Fig. 4b, the trends for NYLD_NUE_ and GYLD/OYLD_NUE_ are displayed, and in Table 3b, the corresponding changes between 1995 and 2021 are given. Results for NUE in PEAS are not shown, because they received only limited N fertilization and were therefore not comparable with other crops in NUE traits. NYLD_NUE_ increased in all crops, but only significant at the 5%-level in WW (8.0%), WR hybrid varieties (13.0%), SW (15.9%) and at the 1%-level in WOSR (20.7%).

### Genotypic, environmental and G × E variation

**Fig. 5:**
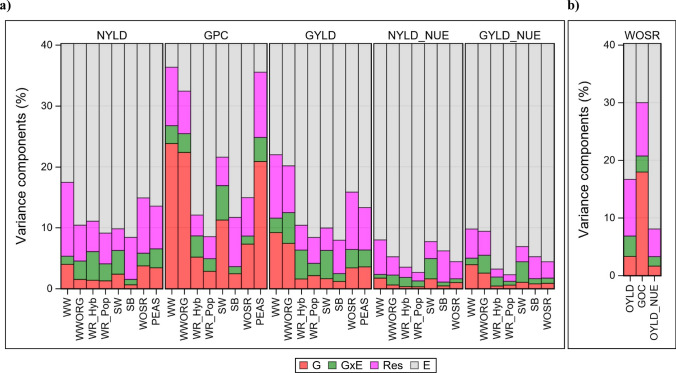
Variance components as percent of marginal variance (total sum of variance components for random effects given by Eq. 1) considering linear genetic trend in the genotype effects and quadratic non-genetic trends in the year effects based on 1995–2021. Traits for OYLD, GOC and OYLD_NUE_ in WOSR are shown in (**b**). Y-axis was truncated at the 40% level. NUE for grain peas were not listed as they received no regular nitrogen fertilizer. *WW* winter wheat, *WWORG* winter wheat under organic treatment regimen, *WR* winter rye,* Hyb* hybrid, *Pop* population varieties, *SW* spring wheat, *SB* spring barley, *WSOR *winter oil seed rape, *PEAS* grain peas, *NYLD* nitrogen yield in grain, *GPC* grain protein concentration, *GOC* grain oil concentration, *GYLD* grain yield, *OYLD* oil yield, *NYLD*_*NUE* (GYLD_NUE_) NUE of nitrogen yield in grain (GYLD_NUE_ = NYLD/available N), *GYLD*_*NUE* (GYLD_NUE_) NUE of grain yield (GYLD_NUE_ = GYLD/available N), *OYLD*_*NUE* (OYLD_NUE_) NUE of oil yield (OYLD_NUE_ = OYLD/available N), available N = N rate + Nmin, *G* genotype, *G* × *E* Genotype ×  environment interaction, *Res* residual, *E* environment

Varieties grown over many years and locations are exposed to a wide range of environmental conditions as shown in Figs. 2 and 3. Variance components for the random effects given in the basic model (Eq. 1) were estimated. The graphical representation of genotypic, G × E interaction, environmental and residual components and the marginal variance is shown in Fig. 5 and more details are given in Supplementary Material Table S1. First, we consider the relative magnitude of variation for individual traits across crops. Environmental variation was the dominating component. For NYLD and GYLD/OYLD it ranged roughly between 80% and 90%, only for GPC/GOC the environmental variation was smaller (60–90%). Nearly the complete variation (around 95%) for NYLD_NUE_ and GYLD/OYLD_NUE_ was caused by the influence of environmental conditions. In contrast to the environmental variation, the genotypic variation was small. For NYLD_NUE_ it was about 1% on average, for GYLD/OYLD_NUE_ about 1.5%, for NYLD about 2.5% and GYLD/OYLD about 4%. Among all traits, the genotypic variation of GPC/GOC was highest, especially for WW, WOSR and PEAS. The G × E component was of about the same magnitude as the genotypic variation. The residual error showed the second largest variation. Across all traits, WW showed the largest and WR hybrid varieties the lowest genotypic variation.

The strikingly low genotypic but high environmental variation for NUE traits compared to the other traits requires an explanation. We think this is due to the fact that NUE is derived as the ratio of two random variables which were approximately independent, e.g., GYLD and available N, which increases the variation according to the law of error propagation (average GYLD per trial and available N were nearly uncorrelated, data not shown). Importantly, GYLD varies between genotypes within one trial, whereas the amount of applied N is the same for all genotypes in the same trial and hence only varies between environments. Further, looking at this ratio in terms of model effects (Eq. 1), we find that the ranking of genotypic effects within a trial is unaffected by the constant value of available N in the denominator, while the environmental effects show an increased variance. This results in a higher percentage of environmental variance in the total variance and hence in lower percentages of the other components as Fig. 5 and Supplementary Material Table S1 show.

Despite the remarkable very large environmental variation, compared to the genotypic component, considerable breeding progress has been achieved as shown in the previous section, because each year new variation due to new varieties enter trials where this variation can be used to achieve progress.

### Genotypic, environmental and G × E correlation

The overall correlation between traits based on observations of variety × year × location × trial series combinations were estimated by marginal correlation coefficients using a univariate approach (Eqs. 7 and 8). We further dissected the marginal correlation coefficient *r*_*M*_ by the genotypic *r*_*G*_, genotype × environment interaction *r*_*G×E*_, residual *r*_*Res*_ and environmental correlation *r*_*E*_ coefficients to show the strength of association of genotypic, environmental and residual effects. To check the plausibility of the marginal correlation, we estimated the Pearson sample correlation coefficient *r*_*P*_ over all observations, which was approximately of the same magnitude as the marginal one. The strength of correlation between traits was denoted by the following categories: |*r*|< 0.15 very weak, 0.15 ≤|*r*|< 0.35 weak, 0.35 ≤|*r*|< 0.55 moderate, 0.55 ≤|*r*|< 0.75 strong, 0.75 ≤|*r*| very strong.

The marginal correlation coefficients *r*_*M*_ in Table 4 show that NYLD was moderate to strong correlated with GYLD/OYLD while NYLD was only weak to moderate correlated with GPC. As expected, GYLD was negative but weak correlated with GPC/GOC. The environmental correlation *r*_*E*_ between NYLD and GYLD/OYLD showed strong to very strong while *r*_*E*_ between NYLD and GPC was less strong in the range of weak to moderate. The genotypic correlation was positive for all crops for NYLD with GYLD/OYLD (*r*_*G*_ = 0.37 on average) and GPC (*r*_*G*_ = 0.45 on average) varying in a wider range than environmental correlation coefficients. This indicates that first, NYLD was stronger linked to GYLD than to GPC/GOC and second that the selection for varieties with high genotypic value for NYLD does not counteract with the selection for varieties with high genotypic value for GYLD/OYLD and GPC. In WOSR, the genotypic correlation for GYLD with GOC (*r*_*G*_ = 0.49) was positive, contrary to the correlation with GPC (*r*_*G*_ = −0.21). However, GPC was negative correlated with GOC (*r*_*G*_ = −0.47).

**Table 4:**
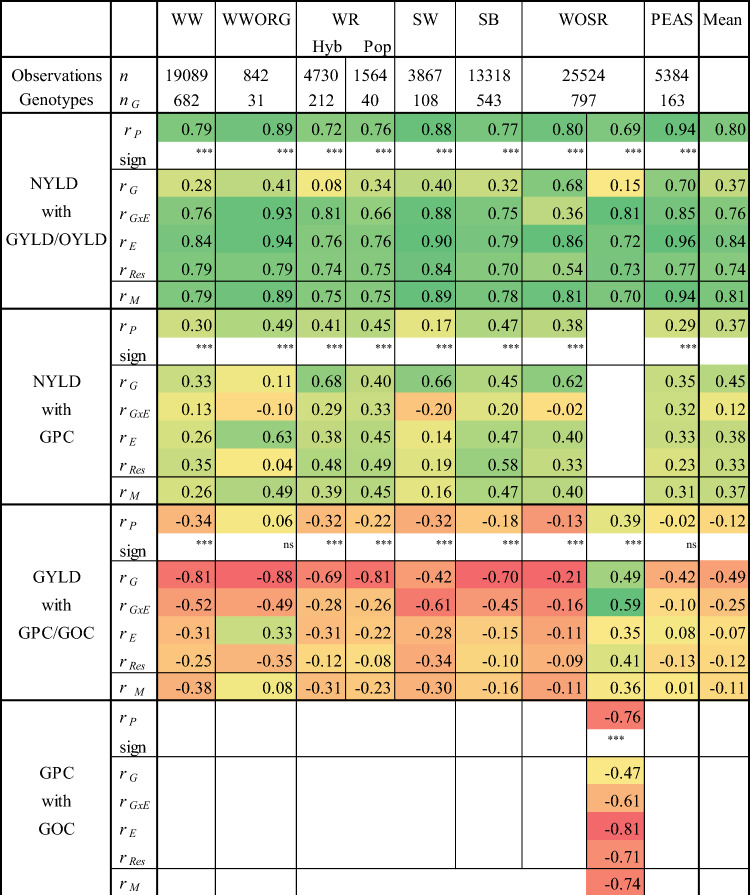
Decomposition of marginal correlation (*r*_*M*_) by genotypic (*r*_*G*_), genotype × environment interaction (*r*_*G×E*_), environmental (*r*_*E*_) and residual (*r*_*Res*_) effects (Eqs. 1 and 7), where *r*_*P*_ is the Pearson sample correlation coefficient, sign its significance level, *n* number ot total observations and *n*_*G*_ number of genotypes. Correlation coefficients are based on years 1995–2021

*WW* winter wheat, *WWORG* winter wheat under organic testing regimen, *WR* winter rye, *Hyb* hybrid varieties, *Pop* population varieties, *WOSR* winter oil seed rape, *SW* spring wheat, *SB* spring barley, *PEAS* grain peas, *NYLD* nitrogen yield in grain, *GPC* grain protein concentration, *GYLD* grain yield, *OYLD* oil yield, *GOC* grain oil concentration, *G* genotype, *Y* year, *L* location, *Y × L × T* interaction of trials within Y × L, *G * × *E* genotype × environment interaction (G × E = G × Y + G × L), *E* environment (E = Y + L + Y × L × T), *Res* residual

To evaluate the question of how strong NYLD was correlated with NYLD_NUE_ and GYLD/OYLD with GYLD/OYLD_NUE_, we also dissected the marginal correlation as described above. For these traits the genotypic, G × E and residual correlations were approximately one (*r*_*G*_ ≈ 1, *r*_*G×E*_ ≈ 1, *r*_*E*_ ≈ 1) while the environmental correlation *r*_*E*_ was lower in the range of 0.33 and 0.83 (Supplementary Material Table S2). This is a general result which holds only if all varieties in an individual trial received the same N rate. The numerical results in Table S2 have been confirmed by a mathematical proof shown in Appendix. In the case of our study, the genotypic value of a variety is about equivalent to the least square estimate of a variety’s 3-year cycle mean using Eq. (1). Hence, the ranks of the estimated variety means for GYLD and GYLD_NUE_, and NYLD and NYLD_NUE_ are about the same. This is demonstrated graphically by a few examples given in Supplementary Material Fig. S1. In consequence, this result indicates that the selection of varieties for high GYLD or high NYLD implies the selection of varieties for high GYLD_NUE_ and NYLD_NUE_.

### Heritability

Traits assessed in registration trials should not only be relevant for evaluating the value for cultivation and use but results obtained during the two- or three-year testing cycle of a variety should also be repeatable at farmers’ fields. As a measure of repeatability, we show heritability coefficients *H*^2^ (Eq. 2a) in Table 2 for individual traits based on the crop-specific testing systems. Further, we show the trial-specific heritability coefficients *q*^2^ (Eq. 2b), which are independent of the number of years and locations for a given crop’s testing cycle.

The total number of trials for a crop’s present testing cycle was in the range of 36 in WOSR and 21 in SW. In the second and third testing year, more overlapping locations were available compared to the other years. PEAS was the only crop with a testing period of 2 years. Across crops, cycle-based heritability was on average across crops highest for GPC/GOC (*H*^2^ = 0.91) followed by GYLD/OYLD (*H*^2^ = 0.78) and NYLD (*H*^2^ = 0.73) while NYLD_NUE_ reached only *H*^2^ = 0.69. Noticeably, the average heritability for GYLD/OYLD_NUE_ (*H*^2^ = 0.75) was higher than for NYLD_NUE_ (*H*^2^ = 0.67). The magnitude of the average trial-based heritability coefficients was of about the same rank order as for the cycle-based coefficients in the range of 0.17 ≤ *q*^2^ ≤ 0.26. Table 2 indicates that across traits, the largest values for cycle-based heritability were achieved in WW, WOSR and PEAS, while heritability in WR hybrid varieties and SB was relatively low. Overall, the average heritability coefficient of NYLD was of about the same magnitude as for GYLD/OYLD, which is generally the most important registration criterion.

## Discussion

### Soil-mineralized nitrogen (Nmin)

When considering the predicted Nmin values as shown in Fig. 2a, it should be noted that the predictive power of the BLUP values was based on a moderate coefficient of determination of *R*^2^ = 41.5% and therefore predicted Nmin values only approximately. Further, the variation between trials is likely lower than the variation for the actual Nmin values as BLUP shrinks toward the mean. We assumed that no noteworthy time trends in Nmin during 1983 and 2021 were present, because registration trials were integrated in the crops typical crop rotation sequence. This is consistent with the fact that in the course of long-term experiments only small net changes in soil-mineralized N were observed (Johnston et al. 2009; Sylvester-Bradley and Kindred 2009; Ladha et al. 2016), because a near steady state may be achieved in fields under continuous crop rotation regimen. In a long-term experiment with winter wheat in the UK, van Grinsven et al. (2022) reported an available nitrogen from soil (nitrogen deposition and natural biological nitrogen fixation from free-living bacteria) between 4 and 64 kg N ha^−1^ with a mean of 30 kg N ha^−1^ showing no trend in time. In an another UK wheat study (Hawkesford and Riche 2020), soil mineral N ranged from 25.6 to 115.7 kg N ha^−1^ between 2006 and 2017. These results indicate a large variation of Nmin, which is in accordance with the distribution of Nmin predicted in this study as shown in Fig. 2b.

### Overall trends for breeding progress of NUE and related traits

Table 3a has shown that N rates decreased considerably. Cassman and Dobermann (2022) reported a reduction of N input in Western and Central Europe during the last decades. They assumed that this was probably due to general agricultural policy and the gradual tightening of fertilizer regulation requirements to reduce nitrate leaching and environmental pollution. We assume that the observed reduction in N rates in registration trials conducted according to good local agronomic practice is also due to the same reasons. SB was the only crop where a significant increase of N rate (20.9%) was found. However, the increasing N rate in SB may be explained by the interaction of several factors. In this crop, N was applied at the rather low malting barley level to balance grain yield and malting quality criteria to fulfill the strict requirements of malting industry (Barmeier et al. 2021). For example, malting barley varieties’ GPC must be below 12% as required by the Federal Plant Variety Office (BSL 2023). We believe that increasing N rates in SB was possible due to the introduction of shorter rh-genotypes without compromising malting quality due to a lower lodging risk. Further, higher N rate increased yield without increasing GPC due to the trade-off mechanism between GYLD and GPC (de Oliveira Silva et al. 2020). This is supported by the fact that GPC did not increase, but decreased by −4.5% as shown in Table 3a.

Despite the strongly reduced N rates, GYLD/OYLD mostly increased significantly, which suggests a breeding progress through new genotypes. Numerous other studies confirmed breeding progress with regard to yield (e.g., Laidig et al. 2014; Mackay et al. 2011; Voss-Fels et al. 2019). Nevertheless, this study shows that breeding progress was even possible under reduced N rates. The reduced GPC in all crops can be attributed to two effects; first to the reduced N fertilizer rates and second to the trade-off effect between yield and protein due to the well-known negative association between both traits. The strong reduction of N fertilizer and consequently the decay in GPC were not reflected by the same magnitude in the changes for NYLD. This can be explained by the stronger link of GYLD with NYLD than of GPC with NYLD, shown by the correlation coefficients in Table 4.

The highest breeding progress for NUE was achieved in WOSR compared with other crops. Despite this progress WOSR still had the lowest NUE levels (e.g., for NYLD_NUE_, 0.25 kg kg^−1^ in 1995 and 0.30 kg kg^−1^ in 2021) among all crops (Table 3, Fig. 4). Our results are in line with Sylvester-Bradley and Kindred (2009) who compared NUE of major agricultural crops in the UK, indicating that harvested WOSR had the lowest NUE, lower than for cereals. WOSR depends on higher N fertilization than other crops due to its low NUE which may partially be explained by the negative correlation between oil and protein (r = −0.76). The breeding focus on high oil concentrations - as one target trait of an oil crop - could conflict with a higher mobilization of N from source to sink. In other studies, reported NUE in WOSR was often not exceeding 60% due to its low ability to remobilize plant stored N (Bouchet et al. 2016; Stahl et al. 2017, 2019).

PEAS are recovering N mostly from air by N_2_ fixation of symbiotic bacteria. Kelstrup et al. (1996) estimated a bacteria fixed N amount of 122 kg N ha^−1^ and a soil accumulated N of 57 kg ha^−1^ achieving a grain yield of about 43 dt ha^−1^ and Ruisi et al. (2012) an average N of 25 kg ha^−1^. We found a higher yield level for PEAS of 50 dt ha^−1^, which may likely be due to the breeding progress of newer varieties investigated in our study. Yang et al. (2017) reported lower GYLD of 21.9–51.5 dt ha^−1^ from six pea varieties in western Canada. Table 3 and Fig. 4 show that PEAS accumulated the highest amount of N in grain besides WW, however, with nearly zero N fertilizer, indicating the effect of a legume crop.

Our study showed a very strong increase in NUE for GYLD/OYLD and a lower one for NYLD. We have to look at this increase by taking two aspects into account. Firstly, the increase due to new improved varieties and secondly, the increase due to the reduction of N fertilizer. Regarding the first aspect, Lassaletta et al. (2014) reported on 50-year NUE trends (% harvested N in protein /N input onto cropland) across cropping systems in Western Europe and found considerable increase in NUE, e.g., in France from about 40% in 1980 to nearly 80% in 2010. Higher NUE of newer varieties in WW was also reported by Guarda et al. (2004), Ladha et al. (2016), Guttieri et al. (2017), Ivic et al. (2021), Sieling and Kage (2021), in WOSR by Stahl et al. (2017, 2019), and in winter triticale by Neuweiler et al. (2022). The second aspect was confirmed by studies testing varieties under different N rates, which found a general agreement that NUE increases with decreasing N rates or, conversely, NUE decreases with increasing available N (Cormier et al. 2016; Sieling and Kage 2021, 2022). However, Cassman and Dobermann (2022) question whether new crop varieties with an apparent improvement of specific traits for NUE were actually generated. They largely attribute the improved NUE in high-fertilizer-use regions to a more judicious use of N fertilizers as a result of policies and regulations aiming to reduce N use, rather than to the benefits of increasing crop yields.

Disentangling the confounded effects of breeding progress by new varieties and the increase of NUE by reduction of N rates was statistically not possible. However, we elaborate in the following that breeding progress for NUE of GYLD was at least as high as for GYLD. Let us assume a constant N level from 1995 to 2021, e.g., in WW (180.1 kg). Then NUE in 1995 is equal to the ratio of GYLD and N level in 1995 (95.4 dt ha^−1^/180.1 kg ha^−1^ ≙ 53.0 kg kg^−1^) and NUE in 2021 is equal to the ratio of GYLD in 2021 and N level in 1995 (103.3 dt ha^−1^/180.1 kg ha^−1^ ≙ 57.4 kg kg^−1^). The derived difference of NUE for GYLD in 2021 and in 1995 (4.4 kg kg^−1^) relative to the NUE level in 1995 (53.0 kg kg^−1^) then equals the relative change of GYLD in WW (4.4 kg kg^−1^/53.0 kg kg^−1^ ≙ 8.3%), both in the hypothetical example and in the observed data (see Table 3a). This calculated 8.3% correspond to the lower limit of breeding progress for NUE, as in reality N levels went down from 1995 to 2021 comprising the actual breeding progress for GYLD and especially NYLD, which would be higher if N levels were maintained at 1995-level. These considerations allow concluding that the breeding progress for NUE of NYLD and GYLD (Table 3a) is actually somewhere in between the breeding progress for NYLD and GYLD (Table 3b) if N rate would have been unchanged at level 1995. Accordingly, breeding progress for NUE was actually achieved by new varieties (under considerable N fertilizer reduction), and improved NUE was not just a result of reduced N fertilizer use over time as Cassman and Dobermann (2022) assumed. Furthermore, the absolute reduction in N rate far outweighed the small reduction of grain accumulated N, indicating considerable mitigation of adverse environmental impacts.

### Genotypic, environmental and G × E variation

Our study built on trial data with a single N rate following good agronomic local practice by providing N according to crop demand. Figure 2 showed that N rates differed strongly between environments, which was most likely due to differences in trial-specific Nmin supply and differences in actual N demand driven by differences in yield potential between trials. Hence, we could not estimate variance components for genotype by N rate interaction. This raises the often-asked question whether trials with only one N level, as in this study, will be efficient enough to select genotypes with high NUE also under lower N fertilizer rates. Most studies on crops’ NUE are conducted with two or more N rates. Cormier et al. (2016) stated in a review paper that numerous studies on wheat (e.g., Ortiz-Monasterio et al. 1997; Le Gouis et al. 2000; Laperche et al. 2006; Barraclough et al. 2010; Cormier et al. 2013), detected significant genotype × N rate interactions for agronomic traits, meaning that the genotypic values of varieties differ between N levels and that selection for varieties in low N target regions may be efficient if the magnitude of genotype × N rate interaction was large compared to the genotypic variation. Also, several studies with contrasting N rates found genotype × N rate interactions for NUE and related traits, but of very low magnitude compared to the genotypic variation, as reported by Voss-Fels et al. (2019), Ivic et al. (2021) and Brasier et al. (2020) in WW, Anbessa et al. (2009) in SB, and Bouchet et al. (2016), Kessel et al. (2012) and Stahl et al. (2017, 2019) in WOSR. Accordingly, results from a UK germplasm diversity trial with recent winter wheat varieties grown under five different N fertilization levels indicated that the ranking of the varieties at each of the N rates is almost identical (Hawkesford and Riche 2020). Further, Hasegawa (2003) and Büchi et al. (2016) argued that crop varieties selected under reduced N rates are not necessarily better adapted to low-input conditions and that breeders should instead devote a majority of their resources to multi-environment testing. These often-observed low genotype × N rate interactions in previous assessments of genotypic variation for NUE indicate that it is rather promising to increase the number of testing environments instead of increasing the number of different N rates when selecting for NUE (Brasier et al. 2020). Registration trials with multiple N rates per trial would presumably provide more insights although one would not expect any differences in the ranking of the varieties regarding genotype-specific N responses. Besides, it would be much more expansive and harder to manage considering the generally large number of varieties and environments in variety testing systems. Accordingly, we expect no different results regarding the variety ranking and respective approval for variety release. From these results, we conclude that a trial system, as evaluated in this study, is at least as efficient to select varieties with high NUE as a system with more N levels, but fewer environments.

The dominating share of environmental variation compared to the genotypic and G × E interaction variation shown in Fig. 5 was in line with results reported by Brasier et al. (2020) in WW, Anbessa et al. (2009) in barley and Stahl et al. (2019) in WOSR. In contrast to these results, Ivic et al. (2021) found that a genotypic variation from a Croatian study in WW varieties developed between 1936 and 2016 for NUE of GYLD which was 42.1% compared to the environmental of only 3.4%. This is a strongly biased result which can be explained by the large trend in breeding progress for NUE between 1936 and 2016, which inflated the genotypic variation. In our study, the genetic trend was taken into account such that we estimated unbiased genotypic variance components (conf. Eq. 1).

Among the cereal crops, WW had the largest and SB the lowest genotypic variation and, vice versa, WW the smallest and SB the largest environmental variation as shown in Fig. 5. This can be ascribed to the fact that in WW varieties with a large spread of baking quality, from fodder to elite types, were included with very different GPC and GYLD (Laidig et al. 2017a). However, it should be pointed out that this large genotypic variation for WW could not be fully used for selection, because genotypes with low GPC have higher yield and are more likely of fodder and not of baking quality whereas the reverse applies for genotypes with lower yield but with higher GPC restricting the range for selection. SB was grown under N rates corresponding to malting barley level to achieve high malting quality. This requires a rather low and well-balanced N fertilization, which is below optimum grain yield. Further, it is known that SB genotypes are not very different, because most of the present malting barley varieties are descendants of “Hana-type” varieties from Moravia in the early 1900s and of the semidwarf variety “Trumpf” in the early 1970s which was likely the reason for this low genotypic variability (Laidig et al. 2017b).

The generally low genotypic variation for NUE and NYLD shown in Fig. 5 raises the question if a further increase of NUE could be achieved by broadening the genotypic variation from a wider germplasm pool outside. The low genotypic variation might have different causes, such as the existence or fixation of unfavorable alleles for NUE in the elite breeding material, or the presence of unfavorable allele combinations which are affecting the highly quantitative trait. Both assumptions could be the result of a low selection pressure on the trait NUE and NYLD in the last decades due to higher fertilization rates. In case of the first assumption that a wider germplasm pool should be considered for future breeding efforts, by broadening the genetic basis of NUE and NYLD. However, the analysis of recent varieties (released and grown before mineral fertilizers were commonly used) or genetic resources like resynthesized lines as source for NUE genetic variation in WOSR was not considered as promising (Kessel et al. 2012). The latter assumption that best combinations of favorable alleles for NUE which already exist in the current elite material have not yet been either produced or found, implicates the need of larger population sizes in breeding programs for NUE to increase the number of meiosis and the chance for selecting best progenies.

This study revealed a strong influence of environmental conditions on total variation of NUE for GYLD/OYLD_NUE_ and NYLD_NUE_ of more than 90%. While trial-specific soil and climate conditions cannot be influenced, improved management including crop rotation, soil tillage and pesticide application may help to improve NUE. Most importantly, optimizing fertilization with regard to distribution, timing, type and amount, under consideration of pre-crop, seasonal weather course and actual crop demand provides substantial potential for increasing NUE in crop production.

### Genotypic, environmental and G × E correlation

The very strong marginal correlation coefficients *r*_*M*_ between NYLD and GYLD/OYLD, shown in Table 4, were in line with results reported by Ivic et al. (2021) and Guttieri et al. (2017) for WW, Anbessa et al. (2009) and Sinebo et al. (2004) for SB and Stahl et al. (2019) for WOSR. This strong marginal correlation between NYLD and GYLD/OYLD confirmed that breeding progress achieved in GYLD/OYLD also resulted in a higher NYLD, while the lower correlation with GPC showed that this trait is of lower influence on NYLD.

The fact that the genotypic correlation coefficient *r*_*G*_ for GYLD/OYLD and NYLD with the corresponding traits for NUE is approximately one, as shown in Supplementary Material Table S2 and confirmed by the proof in Appendix, implies that the rank order of their genotypic values, or approximately equivalent between their least square estimates of variety means, is about the same. In numerous studies strong to very strong correlations coefficients between variety means for GYLD_NUE_ with GYLD were reported indicating this very strong association, however, without mentioning that this is due to the functional relation between trait and its NUE (e.g., Ivic et al. 2021; Muurinen et al. 2006; Stahl et al 2019).

### Heritability

Many studies on NUE were published, however, only a few reported heritability coefficients, but of very different magnitude in the range between 0.25 ≤ *H*^2^ ≤ 0.94, e.g., in WW by Hitz et al. (2017), Ivic et al. (2021), Guttieri et al. (2017) and in SB by Sinebo et al. (2004) and Anbessa et al. (2009). Compared to reported heritability coefficients, we estimated larger values for NUE as shown in Table 2. This spread in reported heritability coefficients may likely be explained, as mentioned previously, by the different sets of genotypes and environments from which they were estimated, which makes it difficult to compare them between studies. So far, NYLD of submitted varieties is not a direct criterion for registration, except in PEAS and in WOSR where protein yield is considered. The fact that the average heritability across crops for NYLD (*H*^2^ = 0.73) was not much lower than for GYLD/OYLD (*H*^2^ = 0.78), which is a very important criterion for registration, indicates that NYLD is about equally reliant as GYLD. Further, weak positive genotypic correlation coefficients between NYLD with GYLD and GPC, as shown in Table 4, give evidence that selection of varieties for high NYLD does not counteract the selection for high GYLD or GPC. For these reasons, we suggest to use NYLD as further criterion in registration trials, or, in the case of WOSR and PEAS where NYLD is already assessed, NYLD should be given a higher weight. Oberforster and Werteker (2005) already advocated for the use of NYLD in Austrian winter wheat registration trials as additional criterion. Reliability of NYLD as registration criterion could be improved if the number of locations at which GPC and NYLD is assessed is increased in WW, WR, SW and SB, beyond the subset of locations where quality samples are taken so far.

## Conclusions

This study assessed breeding progress for NUE and related traits in important crops. We showed that nitrogen fertilization rates in variety trials of cereal crops and winter oilseed rape, except spring barley, were considerably reduced between 1995 and 2021. Despite this reduction, grain and oil yield increased while grain nitrogen yield did not decrease significantly in all crops. NUE for grain yield, oil and nitrogen yield increased strongly, which indicates a large breeding progress for NUE due to improved varieties. However, some of the increase may be attributed to the known effect that NUE increases when nitrogen fertilizer use is reduced. Genotypic variation was low compared to the environmental variation for grain, oil yield and for nitrogen yield, while the environmental variation of NUE for grain and nitrogen yield was even higher. The correlation coefficients showed that grain yield had a stronger influence on nitrogen yield than grain protein concentration. Furthermore, the low positive genotypic correlation of nitrogen yield with grain yield and grain protein concentration suggest that the selection for high nitrogen yield does not counteract with grain yield and grain protein concentration. The result that the ranking of the genotypic values of varieties for grain and nitrogen yield, which is approximately the same as the ranking of the least square estimates for variety means, was the same as for the genotypic values of their corresponding NUE traits, indicates that NUE for grain yield was already taken into account by grain yield as an important registration criterion. Heritability of cycle means for nitrogen yield was only little lower than for grain yield, which means that nitrogen yield is approximately as reliable as grain yield when using it as an additional trait for registration. Therefore, nitrogen yield should be given a higher weight in breeding and variety registration to increase NUE and reduce adverse environmental impact. This study’s results highlight that despite considerable reduction in nitrogen fertilizer inputs in cereal crops and oilseed rape breeding progress was achieved without comprising land use efficiency.

### Electronic supplementary material

Below is the link to the electronic supplementary material.

Supplementary file1 (PDF 67 KB)SM3 Plots between least square means of winter wheat varieties grown in the same cycle

Supplementary file2 (PDF 28 KB)SM1 Variance components for NUE traits and related traits

Supplementary file3 (PDF 27 KB)SM2 Correlation coefficients of NUE traits with related traits

## Data Availability

Data were provided by the Federal Plant Variety Office for exclusive use in this study and are in general not publicly available. Reasonable requests may be addressed to the Federal Plant Variety Office, Hannover, Germany.

## Appendix

### Why genotypic and G × E correlation coefficients for grain yield with NUE for grain yield and nitrogen yield with NUE for nitrogen yield are approximately equal to one

Consider two random variables *W*_*ij*_ and *U*_*ij*_ = *W*_*ij*_/*N*_*j*_, where *W*_*ij*_ = grain yield of *i*th genotype in *j*th environment and *N*_*j*_ = nitrogen input in *j*th environment. For *W*_*ij*_, we assume the random effects model

10 $${W}_{ij}=\mu +{G}_{i}+{L}_{j}+G{L}_{ij}$$

with independent random effects having zero mean and variances ${\text{var}}\left({G}_{i}\right)={\sigma }_{G}^{2}$, ${\text{var}}\left({L}_{j}\right)={\sigma }_{L}^{2}$ and ${\text{var}}\left(G{L}_{ij}\right)={\sigma }_{GL}^{2}$. Here, we will investigate what can be said about the covariance of effects for *W*_*ij*_ and *U*_*ij*_ assuming model (10) for *W*_*ij*_. For simplicity, we will assume that *N*_*j*_ is independent of all effects in the model for *W*_*ij*_. This could be relaxed by assuming a covariance with *L*_*j*_, but this is not expected to have any substantive bearing on the subsequent derivation, as will be explained briefly at the end. The model for *U*_*ij*_ can be written as

11 $${U}_{ij}=\frac{\mu +{G}_{i}+{L}_{j}+G{L}_{ij}}{{N}_{j}}={\mu }_{j}^{\mathrm{^{\prime}}}+{G}_{ij}^{\mathrm{^{\prime}}}+{L}_{j}^{\mathrm{^{\prime}}}+{GL}_{ij}^{\mathrm{^{\prime}}}$$

where $${\mu }_{j}^{\mathrm{^{\prime}}}=\frac{\mu }{{N}_{j}}$$, $${G}_{ij}^{\mathrm{^{\prime}}}=\frac{{G}_{i}}{{N}_{j}}$$, $${L}_{j}^{\mathrm{^{\prime}}}=\frac{{L}_{j}}{{N}_{j}}$$ and $${GL}_{ij}^{\mathrm{^{\prime}}}=\frac{G{L}_{ij}}{{N}_{j}}$$.

A challenge in the subsequent derivation is that all effects in (11) involve the random variable *N*_*j*_, hence we need to check if there are correlations among the effects. It will be useful to make use of the laws of total variance and total covariance (Rudary 2009). Let *X*, *Y* and *Z* be three random variables. Then the law of total variance states that

$${\text{var}}\left(Y\right)=E\left[{\text{var}}\left(\left.Y\right|X\right)\right]+{\text{var}}\left[E\left(\left.Y\right|X\right)\right].$$

The law of total covariance states that

$${\text{cov}}\left(X,Y\right)=E\left[{\text{cov}}\left(\left.X,Y\right|Z\right)\right]+{\text{cov}}\left[E\left(\left.X\right|Z\right),E\left(\left.Y\right|Z\right)\right].$$

If we identify *Z* with *N*_*j*_, and *X* and *Y* with any of the two effects in (11), then it emerges from the law of total covariance that the effects in (11) must all be uncorrelated from one another. For example, we find that

$${\text{cov}}\left({G}_{ij}^{\mathrm{^{\prime}}},{L}_{j}^{\mathrm{^{\prime}}}\right)=E\left[{\text{cov}}\left(\left.{G}_{ij}^{\mathrm{^{\prime}}},{L}_{j}^{\mathrm{^{\prime}}}\right|{N}_{j}\right)\right]+{\text{cov}}\left[E\left(\left.{G}_{ij}^{\mathrm{^{\prime}}}\right|{N}_{j}\right),E\left(\left.{L}_{j}^{\mathrm{^{\prime}}}\right|{N}_{j}\right)\right]=$$

$$E\left[\frac{1}{{N}_{j}^{2}}{\text{cov}}\left({G}_{i},{L}_{j}\right)\right]+{\text{cov}}\left[\mathrm{0,0}\right]=0$$

Before we consider covariances among effects for *W*_*ij*_ and *U*_*ij*_, we need to transition (11) into a model of the same form as (10). To do so, we may define

$${\mu }_{j}^{\mathrm{^{\prime}}}={\mu }^{^{\prime\prime} }+{F}_{j}^{^{\prime\prime} },$$

where ${\mu }^{^{\prime\prime} }=E\left({\mu }_{j}^{^{\prime} }\right)$ and ${F}_{j}^{^{\prime\prime} }={\mu }_{j}^{\mathrm{^{\prime}}}-{\mu }^{^{\prime\prime} }$. Similarly, we define

$${G}_{ij}^{\mathrm{^{\prime}}}={G}_{i}^{^{\prime\prime} }+{H}_{ij}^{^{\prime\prime} },$$

where ${G}_{i}^{^{\prime\prime} }=E\left(\left.{G}_{ij}^{\mathrm{^{\prime}}}\right|{G}_{i}\right)$ and ${H}_{ij}^{^{\prime\prime} }={G}_{ij}^{\mathrm{^{\prime}}}-E\left(\left.{G}_{ij}^{\mathrm{^{\prime}}}\right|{G}_{i}\right)$. With these definitions, we can rewrite (11) as

12 $${W}_{ij}={\mu }^{^{\prime\prime} }+{G}_{i}^{^{\prime\prime} }+{L}_{j}^{^{\prime\prime} }+{GL}_{ij}^{^{\prime\prime} },$$

where ${L}_{j}^{^{\prime\prime} }={L}_{j}^{\mathrm{^{\prime}}}+{F}_{j}^{^{\prime\prime} }$ and ${GL}_{ij}^{^{\prime\prime} }={GL}_{ij}^{\mathrm{^{\prime}}}+{H}_{ij}^{^{\prime\prime} }$. With this reparameterization, we can now study covariances among effects between models (10) and (12), using the law of total covariance and the delta method (Johnson et al. 1993).

(i) $${\text{cov}}\left({G}_{i},{G}_{i}^{^{\prime\prime} }\right)=E\left[\left.{\text{cov}}\left({G}_{i},{G}_{i}^{^{\prime\prime} }\right)\right|{N}_{j}\right]+{\text{cov}}\left[E\left(\left.{G}_{i}\right|{N}_{j}\right),E\left(\left.{G}_{i}^{^{\prime\prime} }\right|{N}_{j}\right)\right]\approx \frac{{\sigma }_{G}^{2}}{{\varphi }_{N}}$$
(ii) $${\text{cov}}\left({L}_{j},{L}_{j}^{^{\prime\prime} }\right)=E\left[\left.{\text{cov}}\left({L}_{j},{L}_{j}^{^{\prime\prime} }\right)\right|{N}_{j}\right]+{\text{cov}}\left[E\left(\left.{L}_{j}\right|{N}_{j}\right),E\left(\left.{L}_{j}^{^{\prime\prime} }\right|{N}_{j}\right)\right]\approx \frac{{\sigma }_{L}^{2}}{{\varphi }_{N}}$$
(iii) $${\text{cov}}\left(G{L}_{ij},{GL}_{ij}^{^{\prime\prime} }\right)=E\left[\left.{\text{cov}}\left(G{L}_{ij},{GL}_{ij}^{^{\prime\prime} }\right)\right|{N}_{j}\right]+{\text{cov}}\left[E\left(\left.G{L}_{ij}\right|{N}_{j}\right),E\left(\left.{GL}_{ij}^{^{\prime\prime} }\right|{N}_{j}\right)\right]\approx \frac{{\sigma }_{GL}^{2}}{{\varphi }_{N}}$$

where ${\varphi }_{N}=E\left({N}_{j}\right)$. Similarly, the variances of the random effects in (12) can be approximated as follows:

(i) $${\text{var}}\left({G}_{i}^{^{\prime\prime} }\right)\approx \frac{{\left[E\left({G}_{i}\right)\right]}^{2}}{{\varphi }_{N}^{4}}{\sigma }_{N}^{2}+\frac{{\sigma }_{G}^{2}}{{\varphi }_{N}^{2}}=\frac{{\sigma }_{G}^{2}}{{\varphi }_{N}^{2}}$$
(ii) $${\text{var}}\left({L}_{j}^{^{\prime\prime} }\right)={\text{var}}\left({L}_{j}^{\mathrm{^{\prime}}}+{F}_{j}^{^{\prime\prime} }\right)\approx \frac{{\sigma }_{L}^{2}}{{\varphi }_{N}^{2}}+\frac{{\mu }^{2}{\sigma }_{N}^{2}}{{\varphi }_{N}^{4}}$$
(iii) $${\text{var}}\left({GL}_{ij}^{^{\prime\prime} }\right)={\text{var}}\left({GL}_{ij}^{\mathrm{^{\prime}}}+{G}_{ij}^{\mathrm{^{\prime}}}-E\left(\left.{G}_{ij}^{\mathrm{^{\prime}}}\right|{G}_{i}\right)\right)\approx \frac{{\sigma }_{GL}^{2}}{{\varphi }_{N}^{2}}+{\text{var}}\left[{G}_{i}\left(\frac{1}{{N}_{j}}-\frac{1}{{\varphi }_{N}}\right)\right]\approx \frac{{\sigma }_{GL}^{2}}{{\varphi }_{N}^{2}}$$

where ${\sigma }_{N}^{2}={\text{var}}\left({N}_{j}\right)$. From these results, the correlations can be approximated as

(i) $${\text{corr}}\left({G}_{i},{G}_{i}^{^{\prime\prime} }\right)\approx 1$$
(ii) $${\text{corr}}\left({L}_{j},{L}_{j}^{^{\prime\prime} }\right)\approx \frac{1}{\sqrt{\left(1+\frac{{\mu }^{2}{\sigma }_{N}^{2}}{{\varphi }_{N}^{2}{\sigma }_{L}^{2}}\right)}}<1$$
(iii) $${\text{corr}}\left(G{L}_{ij},{GL}_{ij}^{^{\prime\prime} }\right)\approx 1$$

As noted before, we have assumed here that *L*_*j*_ and *N*_*j*_ are uncorrelated. If a covariance is allowed, this will only affect the correlation ${\text{corr}}\left({L}_{j},{L}_{j}^{^{\prime\prime} }\right)$, but it will still be the case that ${\text{corr}}\left({L}_{j},{L}_{j}^{^{\prime\prime} }\right)<1$.

## Abbreviations

GOC: Grain oil concentration
GPC: Grain protein concentration
GYLD: Grain yield
GYLD_NUE_: NUE of grain yield
Nmin: Soil-mineralized nitrogen
NUE: Nitrogen use efficiency
NYLD: Nitrogen yield in grain
NYLD_NUE_: NUE of nitrogen yield
OYLD: Oil yield
PEAS: Grain peas
SB: Spring barley
SLF: Soil fertility
SW: Spring wheat
WOSR: Winter oil seed rape
WR: Winter rye
WW: Winter wheat
WWORG: Winter wheat organic regimen
