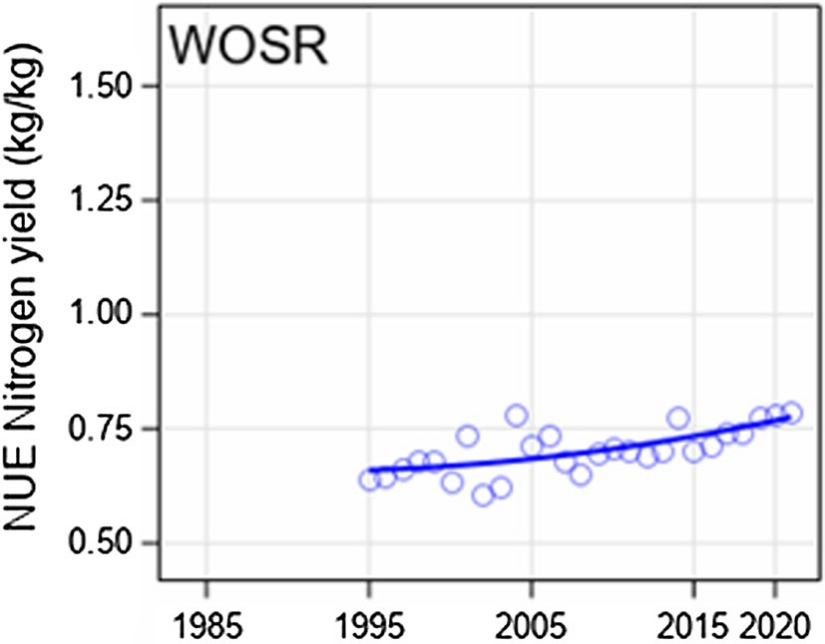# Correction to: Breeding progress of nitrogen use efficiency of cereal crops, winter oilseed rape and peas in long-term variety trials

**DOI:** 10.1007/s00122-024-04630-z

**Published:** 2024-06-10

**Authors:** F. Laidig, T. Feike, C. Lichthardt, A. Schierholt, H. P. Piepho

**Affiliations:** 1 Institute of Crop Science, Biostatistics Unit, University of Hohenheim, Fruwirthstrasse 23, 70599 Stuttgart, Germany; 2 Julius Kühn Institute – Federal Research Centre for Cultivated Plants, Institute for Strategies and Technology Assessment, Stahnsdorfer Damm 81, 14532 Kleinmachnow, Germany; 3 Bundessortenamt, Osterfelddamm 60, 30627 Hannover, Germany; 4 Plant Breeding Methodology, Georg-August-University Göttingen, Carl-Sprengel-Weg 1, 37075 Göttingen, Germany

**Correction to: Theoretical and Applied Genetics (2024) 137:45.** 10.1007/s00122-023-04521-9

The overall trends of winter oilseed rape (WOSR) for nitrogen yield and NUE of nitrogen yield in Table 3 and in Fig. 4 are erroneous. The authors are grateful to Christian Flachenecker, Oilseed rape breeder, Norddeutsche Pflanzenzucht, Hohenlieth, for pointing to this error. We apologize for the mistake. The paper should be modified as follows.

On page 4 after the sentence “As only GPC was available in the dataset, nitrogen yield (NYLD) was calculated as N in kg ha^−1^ accumulated in grain by NYLD = 100 × GYLD × (DM/100) × [(GPC/100)/c], where DM is the percent dry matter content in grain and c is the protein equivalent factor,” the following sentences should be added: “For WOSR, GPC and GYLD are given as 91% DM and for PEAS as 86% DM. For WOSR and PEAS NYLD is calculated as NYLD = 100 × GYLD × GPC/c.”

On page 8 the sentence “We found the highest NYLD in WW and PEAS whereas WOSR had a rather low NYLD (about 51 kg ha^−1^).” should be changed to “We found the highest NYLD in WW and PEAS and the lowest in SB (94.3 kg ha^−1^).”

In Table 3a, row “Winter oil seed rape” for “Nitrogen yield kg ha^−1^” in colums 1995, 2021, Diff, % and Sign the figures should be read as 131.4, 134.6, 0.1, 0.1 and ^ns^, respectively. In Table 3b, row “Winter oil seed rape” for “NUE Nitrogen yield kg kg^−1^”, in columns 1995, 2021, Diff, % and Sign the figures should be read as 0.66, 0.77, 0.12, 17.9 and ^***^, respectively.

Figure 4a in row panel “Nitrogen (kg/ha)” the plot for WOSR should be replaced by this plot:
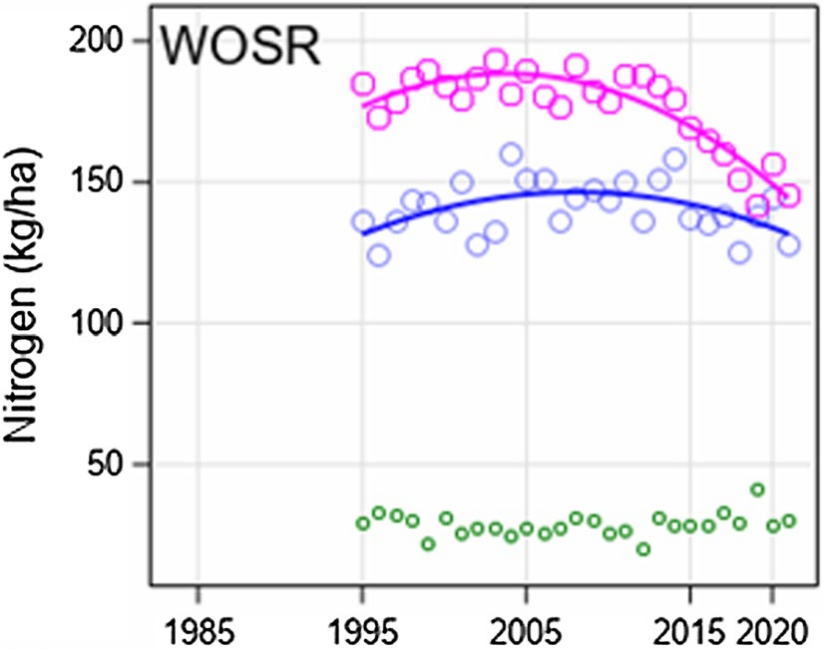


In Fig. 4b in row panel “NUE Grain/Oil yield (kg/kg)” the plot for WOSR should be replaced by this plot: