# Supplementary material for: Breeding progress of nitrogen use efficiency of cereal crops, winter oilseed rape and peas in long-term variety trials
Source: Theor Appl Genet. 2024 Feb 8;137(2):45. doi: 10.1007/s00122-023-04521-9 (PMC10853085; doi:10.1007/s00122-023-04521-9)
Supplement: Supplementary file 1 — Supplementary file1 (PDF 67 KB)SM3 Plots between least square means of winter wheat varieties grown in the same cycle [file 122_2023_4521_MOESM1_ESM.pdf]

Supplementary Material SM3

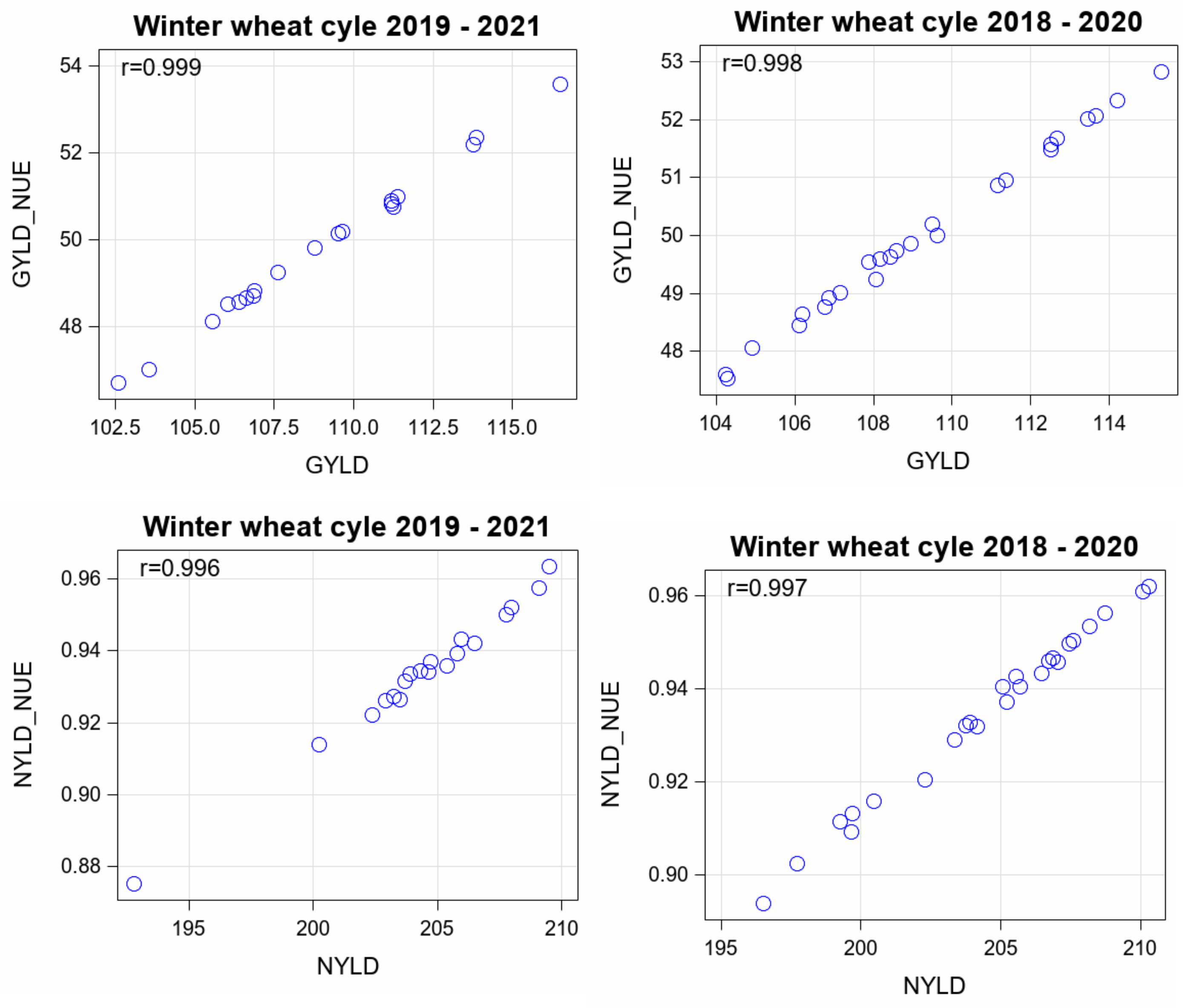

Fig. S1 Correlation between least square means of winter wheat varieties grown in the same cycle. Plots are demonstrating that rank order of means for NYLD and GYLD with the corresponding means of the NUE traits are approximately the same.

NYLD Nitrogen yield in grain; *GYLD* Grain yield; *NYLD_NUE* NUE of nitrogen yield; *GYLD_NUE* NUE of grain yield;
