# Supplementary material for: Breeding progress of nitrogen use efficiency of cereal crops, winter oilseed rape and peas in long-term variety trials
Source: Theor Appl Genet. 2024 Feb 8;137(2):45. doi: 10.1007/s00122-023-04521-9 (PMC10853085; doi:10.1007/s00122-023-04521-9)
Supplement: Supplementary file 2 — Supplementary file2 (PDF 28 KB)SM1 Variance components for NUE traits and related traits [file 122_2023_4521_MOESM2_ESM.pdf]

Table S1 Variance components as percent of total sum of components using Eq. (1) based on years 1995–2021. NUE for grain peas were not listed as they received no regular nitrogen fertilizer.

NYLD	WW	WWORG	RW_Hyb	RW_Pop	SW	SB	WOSR	PEAS	Mean
G	4.0	1.5	1.4	1.3	2.4	0.6	3.7	3.4	2.3
GxY	0.9	1.6	1.4	0.5	1.0	0.4	1.1	1.2	1.0
GxL	0.4	1.5	3.3	2.3	2.9	0.5	1.0	2.0	1.7
Res	12.1	5.9	5.0	5.0	3.6	6.9	9.1	7.0	6.8
Y	9.1	0.0	3.8	3.2	13.2	5.3	9.6	9.9	6.8
L	18.3	23.9	33.3	35.0	31.5	24.7	27.1	25.7	27.4
YxLxT	55.1	65.7	51.8	52.7	45.5	61.6	48.3	50.9	53.9
GxE	1.3	3.0	4.7	2.8	3.9	0.9	2.1	3.1	2.7
E	82.5	89.6	88.9	90.9	90.2	91.6	85.1	86.4	88.2

GPC/GOC	WW	WWORG	RW_Hyb	RW_Pop	SW	SB	WOSR GPC	WOSR GOC	PEAS	Mean
G	23.8	22.4	5.2	2.8	11.3	2.5	7.3	18.0	20.9	12.7
GxY	1.6	1.8	1.1	0.9	1.7	0.9	0.7	1.9	2.4	1.4
GxL	1.3	1.3	2.4	1.2	3.9	0.2	0.6	0.9	1.5	1.5
Res	9.6	7.0	3.4	3.6	4.7	8.1	6.3	9.3	10.7	7.0
Y	7.0	8.3	11.3	11.7	10.3	25.8	10.8	12.6	13.3	12.3
L	19.3	5.7	18.8	19.3	16.1	7.3	26.0	18.7	10.3	15.7
YxLxT	37.3	53.6	57.8	60.5	52.0	55.1	48.3	38.7	40.8	49.4
GxE	2.9	3.1	3.5	2.1	5.7	1.2	1.3	2.8	4.0	2.9
E	63.6	67.6	87.9	91.5	78.4	88.3	85.1	70.0	64.5	77.4

GYLD/OYLD	WW	WWORG	RW_Hyb	RW_Pop	SW	SB	WOSR GYLD	WOSR OYLD	PEAS	Mean
G	9.2	7.4	1.6	2.1	1.6	1.2	3.4	3.3	3.6	3.7
GxY	1.5	2.5	1.4	0.6	1.3	0.5	1.6	1.8	1.2	1.4
GxL	0.9	2.5	3.3	1.4	3.4	0.8	1.4	1.7	1.6	1.9
Res	10.4	7.7	4.1	4.2	3.7	5.5	9.4	9.8	7.0	6.9
Y	14.8	0.0	11.4	9.2	15.0	14.4	18.3	19.7	13.5	12.9
L	23.1	25.5	32.3	33.3	30.5	24.5	20.1	16.6	20.2	25.1
YxLxT	40.1	54.4	46.0	49.1	44.6	53.1	45.8	47.0	53.0	48.1
GxE	2.4	5.0	4.7	2.0	4.6	1.3	3.0	3.5	2.8	3.3
E	78.0	79.8	89.6	91.6	90.0	92.0	84.1	83.3	86.7	86.1

$NYLD_{NUE}$	WW	WWORG	RW_Hyb	RW_Pop	SW	SB	WOSR	Mean
G	1.7	0.6	0.3	0.3	1.6	0.5	1.0	0.9
GxY	0.4	0.7	0.5	0.1	0.9	0.3	0.3	0.7
GxL	0.2	0.9	1.0	0.8	2.4	0.3	0.3	0.7
Res	5.7	3.0	1.7	1.4	2.8	5.1	2.8	2.0
Y	4.9	1.0	1.6	2.2	18.3	3.9	4.1	4.2
L	40.8	67.3	30.3	23.3	25.2	36.9	15.8	19.7
YxLxT	46.4	26.4	64.6	71.7	48.7	53.0	75.7	44.7
GxE	0.6	1.6	1.5	1.0	3.3	0.6	0.6	1.3
E	92.0	94.8	96.5	97.3	92.3	93.8	95.6	94.6

$GYLD/OYLD_{NUE}$	WW	WWORG	RW_Hyb	RW_Pop	SW	SB	WOSR GYLD	WOSR OYLD	Mean
G	3.9	2.6	0.4	0.6	1.0	0.8	0.9	1.6	1.5
GxY	0.6	1.1	0.4	0.1	0.9	0.3	0.4	0.8	0.6
GxL	0.4	1.8	1.1	0.5	2.5	0.5	0.4	0.8	1.0
Res	4.8	3.9	1.3	1.1	2.5	3.6	2.7	4.8	3.1
Y	6.2	0.0	0.3	0.3	14.5	12.8	4.5	5.1	5.5
L	45.2	70.6	34.5	28.8	28.4	38.6	14.6	25.6	35.8
YxLxT	38.7	20.0	62.0	68.6	50.2	43.4	76.4	61.3	52.6
GxE	1.1	2.9	1.5	0.6	3.4	0.9	0.9	1.6	1.6
E	90.2	90.6	96.8	97.7	93.1	94.8	95.6	91.9	93.8

WW Winter wheat; *WWORG* Winter wheat under organic testing regimen; *WR* Winter rye, *Hyb* hybrid and *Pop* population varieties; *SW* spring wheat; *SB* Spring barley; *WSOR* Winter oil seed rape; *PEAS* Grain peas; *NYLD* Nitrogen yield in grain; *GPC* Grain protein concentration; *GOC* Grain oil concentration; *GYLD* Grain yield; *OYLD* Oil yield; $GYLD_{NUE}$ Nitrogen use efficiency of nitrogen yield in grain ($GYLD_{NUE}$ = NYLD/N avail); $GYLD_{NUE}$ Nitrogen use efficiency of grain yield ($GYLD_{NUE}$ = GYLD/N avail); $OYLD_{NUE}$ Nitrogen use efficiency of oil yield ($OYLD_{NUE}$ = OYLD/N avail); *N avail* : N rate + Nmin; *G* Genotype; *Y* Year; *L* Location; *Y×L×T* Interaction of trials within Y×L; *Res* Residual; *G × E* Genotype × environment (G×E= G×Y+G×L); *E* Environment (E=Y+L+Y×L×T);
