# Supplementary material for: Breeding progress of nitrogen use efficiency of cereal crops, winter oilseed rape and peas in long-term variety trials
Source: Theor Appl Genet. 2024 Feb 8;137(2):45. doi: 10.1007/s00122-023-04521-9 (PMC10853085; doi:10.1007/s00122-023-04521-9)
Supplement: Supplementary file 3 — Supplementary file3 (PDF 27 KB)SM2 Correlation coefficients of NUE traits with related traits [file 122_2023_4521_MOESM3_ESM.pdf]

Table S2 Decomposition of marginal correlation (r_M) by genotypic (r_G), genotype × environment interaction ($r_{G \times E}$), environmental (r_E) and residual (r_{Res}) effects, where r_p is the Pearson sample correlation coefficient, sign its significance level, n number ot total observations and n_G number of genotypes. Correlation coefficients are based on years 1995–2021. NUE for grain peas were not listed as they received no regular nitrogen fertilizer.

		WW	WWORG	WR Hyb	WR Pop	SW	SB	WOSR	Mean
Observations	n	19089	842	4730	1564	3867	13318	21564	
Genotypes	n_G	682	31	212	40	108	543	797	
NYLD with $NYLD_{NUE}$	r_P	0.61	0.52	0.41	0.49	0.62	0.83	0.35	0.55
	sign	***	***	***	***	***	***	***	
	r_G	1.00	1.03	1.01	1.02	1.00	1.00	1.00	1.01
	r_{GxE}	0.98	0.93	1.00	0.88	0.98	0.99	0.93	0.96
	r_E	0.59	0.48	0.42	0.44	0.68	0.83	0.33	0.54
	r_{Res}	0.98	0.93	0.89	0.96	0.98	0.99	0.92	0.95
	r_M	0.63	0.51	0.44	0.46	0.70	0.84	0.37	0.57
GYLD with $GYLD_{NUE}$	r_P	0.63	0.41	0.45	0.46	0.66	0.81	0.35	0.54
	sign	***	***	***	***	***	***	***	
	r_G	1.00	not est	0.99	not est	1.00	1.00	1.00	1.00
	r_{GxE}	0.97	not est	0.96	not est	0.99	0.99	0.96	0.97
	r_E	0.61	not est	0.41	not est	0.71	0.80	0.30	0.57
	r_{Res}	0.98	not est	0.93	not est	0.98	0.99	0.92	0.96
	r_M	0.65	not est	0.44	not est	0.73	0.81	0.35	0.60
OYLD with $OYLD_{NUE}$	r_P							0.56	
	sign							***	
	r_G							0.99	
	r_{GxE}							0.97	
	r_E							0.51	
	r_{Res}							0.96	
	r_M							0.56	

WW Winter wheat; *WWORG* Winter wheat under organic testing regimen; *WR* Winter rye; *Hyb* Hybrid varieties; *Pop* Population varieties *WOSR* Winter oil seed rape; *SW* spring wheat; *SB* Spring barley; *PEAS* Grain peas; *NYLD* Nitrogen yield in grain; *GPC* Grain protein concentration; *GYLD* Grain yield; *OYLD* Oil yield; *GOC* Grain oil concentration; $NYLD_{NUE}$ Nitrogen use efficiency for nitrogen yield in grain; $GYLD_{NUE}$ Nitrogen use efficiency for grain yield; $OYLD_{NUE}$ Nitrogen use efficiency for oil yield; *not est* not estimable; *G* Genotype; *Y* Year; *L* Location; *Y×L×T* Interaction of trials within Y×L; *G × E* Genotype × environment interaction (G×E= G×Y+G×L); *E* Environment (E=Y+L+Y×L×T); *Res* Residual;
